# Plant crude extracts containing oligomeric hemagglutinins protect chickens against highly Pathogenic Avian Influenza Virus after one dose of immunization

**DOI:** 10.1007/s11259-022-09942-3

**Published:** 2022-05-28

**Authors:** Hoang Trong Phan, Hanh Xuan Tran, Thuong Thi Ho, Van Thi Pham, Vy Thai Trinh, Tra Thi Nguyen, Ngoc Bich Pham, Ha Hoang Chu, Udo Conrad

**Affiliations:** 1 Leibniz Institute of Plant Genetics and Crop Plant Research (IPK), Gatersleben, Germany; 2 JSC Central Veterinary NAVETCO, 29A Nguyen Dinh Chieu, 1 District, Ho Chi Minh, Vietnam; 3 Institute of Biotechnology, Vietnam Academy of Science and Technology, 18-Hoang Quoc Viet, Cau Giay, Ha Noi, Vietnam; 4 Graduate University of Science and Technology, Vietnam Academy of Science and Technology, 18-Hoang Quoc Viet, Cau Giay, Ha Noi, Vietnam

**Keywords:** Plant-based vaccine, Hemagglutinin oligomers, Avian flu, Challenge experiments

## Abstract

Highly pathogenic avian influenza viruses (HPAIV) have been responsible for causing several severe outbreaks across the world. To protect poultry farms and to prevent the possible spread of new influenza pandemics, vaccines that are both efficacious and low-cost are in high demand. We produced stable, large hemagglutinin H5 oligomers *in planta* by the specific interaction between S•Tag and S•Protein*.* H5 oligomers combined via S•Tag::S•Protein interaction in plant crude extracts induced strong humoral immune responses, strong neutralizing antibody responses, and resistance in chickens after challenge with a wild type HPAIV H5 virus strain. In all three parameters, plant crude extracts with H5 oligomers induced better responses than crude extracts containing trimers. The neutralizing antibodies induced by by two-dose and one dose immunization with an adjuvanted crude extract containing H5 oligomer protected vaccinated chickens from two lethal H5N1 virus strains with the efficiency of 92% and 100%, respectively. Following housing vaccinated chickens together with ten non-immunized chickens, only one of these chickens had detectable levels of the H5N1 virus. To facilitate the easy storage of a candidate vaccine, the H5 oligomer crude extracts were mixed with adjuvants and stored for 3.5 and 5.5 months at 4 °C, and chickens were immunized with these crude extracts. All these vaccinated chickens survived after a lethal H5N1 virus challenge. H5 oligomer crude extracts are comparable to commercial vaccines as they also induce strong virus-neutralizing immune responses following the administration of a single dose. The cost-effective production of plant crude extract vaccine candidates and the high stability after long-term storage will enable and encourage the further exploration of this technology for veterinary vaccine development.

**Supplementary Information:**

The online version contains supplementary material available at 10.1007/s11259-022-09942-3.

## Introduction

The starting point of vaccinology was the development of a smallpox vaccine by Edward Jenner more than 200 years ago. Vaccination has been successfully applied to fight against infectious diseases in humans as well as for veterinary purposes (Andre 2003). A vaccine should be highly efficacious, easily to be employed, and should have additional benefits. These benefits can include high productivity for competition with extended possibilities for disease management. The development of effective plant-based bioreactors has reinforced the idea that the application of this technology for veterinary vaccine production is feasible (Kolotilin et al. 2014; Takeyama et al. 2015). Subunit vaccines from plants, which share low production and infrastructure cost with ease of scale-up and high stability are of special interest (Topp et al. 2016). Transient expression by agroinfiltration of tobacco plants has been developed and applied in several cases as a general, fast and reliable technology for the production of therapeutic proteins *in planta* (Chen et al. 2013).

Zoonotic pathogens are a common and recurring source of infectious diseases in humans (Woolhouse and Gowtage-Sequeria 2005). Avian flu is an example of a zoonotic disease and it is expected to cause global pandemics because it is relatively easily spread by birds and can directly infect humans (Yen and Webster 2009). It belongs to the group of Influenza A viruses (negative-stranded enveloped orthomyxoviruses), which cause severe and often fatal respiratory illnesses (Cox et al. 2004). Such HPAIV caused several severe outbreaks in South-East Asia, Europe, USA, and Canada in recent years (European Centre for Disease Prevention and Control 2020).

Efficacious and low-cost vaccines are needed for the protection of poultry and the prevention of a new influenza pandemic. Production of plant-based vaccines, especially using transient expression is considered a promising method for generating safe, fast, stable, and low-cost vaccines (Topp et al. 2016; for a review see Chen et al. 2013). Several plant-based vaccine candidates against Influenza A viruses were produced in the tobacco species *N. benthamiana* by agroinfiltration (D’Aoust et al. 2008; Landry et al. 2010). These plant-based vaccines required downstream processing methods including filtration, diafiltration, continuous low centrifugation, and tangential flow filtration and/or chromatographic steps for production (D’Aoust et al. 2008; Landry et al. 2010). An Influenza A (H1N1) swine flu vaccine has been developed in plants by Medicago (https://www.bioprocessonline.com/doc/medicago-s-innovative-vaccine-production-using-tobacco-plants-0001). Recently, a plant-based COVID-19 vaccine developed by Medicago has been approved in Canada (https://www.cbc.ca/news/health/medicago-s-homegrown-plant-based-covid-19-vaccine-approved-by-health-canada-1.6362745). However, various constraints such as high downstream cost and/or low expression levels are in contradiction to the need for economical production of vaccines for veterinary application. Therefore, a general goal of this project was the combination of stably high expression of vaccine candidates, high immunogenicity, and minimization of downstream cost. For these purposes, we produced trimers of avian flu hemagglutinin in plants, purified them, and presented by hemagglutination inhibition assays, that these trimers can induce neutralizing immune responses in mice (Phan et al. 2013). Such purified hemagglutinin (H5) trimers from transiently transformed *N. benthamiana* plants were tested in a severe HPAIV H5N1 challenge experiment with chickens (Phan et al. 2013). Chickens immunized with purified trimers were fully protected (Phan et al. 2013). Furthermore, H5 trimers were combined by an *in planta* approach via disulfide bonds, homoantiparallel peptides, or homodimer proteins to H5 oligomers (Phan et al. 2020). Plant crude extracts with oligomers induced neutralizing antibodies in vaccinated mice (Phan et al. 2020). In a further challenge experiment, eleven of twelve chickens (92%) vaccinated with adjuvanted H5 oligomer crude extracts were protected from lethal disease whereas nine out of twelve chickens (75%) vaccinated with adjuvanted H5 trimer crude extracts survived (Phan et al. 2020). Recently, we created another novel way to produce stable and large hemagglutinin H5 (from strain A/duckViet Nam/TG24-01/2005 (H5N1), belonging to clade 1.1) oligomers *in planta* by the specific interaction between S•Tag and S•Protein (Phan et al. 2017)*.* The high-affinity interaction between S•Protein and S•·Tag of bovine pancreatic ribonuclease A was reported as a means for protein purification (Raines et al. 2000) or pharmaceutical purposes as delivery of drugs (Backer et al. 2002). The transient co-expression of H5 trimers with a S•Tag and S•Protein-TPs, multimerized by disulfide bonds via cysteine residues in C-terminally fused tailpiece sequences led to to the formation of very large oligomers in the endoplasmic reticulum of plant cells (Phan et al. 2017). These crude extracts with H5 oligomers showed high hemagglutination titers and induced specific neutralizing antibodies in mice documented by high hemagglutination inhibition titers (Phan et al. 2017). These results opened the way to using crude extracts and avoiding expensive affinity purifications (Phan et al. 2017). However, in the previous study, the immunogenicity of H5 oligomers and H5-S•Tag trimers were not evaluated in chicken via virus challenge experiments.

In the present study, we analyzed, if this strategy could also be applied for other H5 variants (from H5N1 A/Chicken/DL/NAVET_0292/2013 strain) belonging to clade 2.3.2.1c which is the currently circulating clade in Vietnam. Moreover, we evaluated if crude extracts with large H5 oligomers based on S•Tag::S•Protein interaction could induce neutralizing immune responses in chickens tested in virus challenge experiments. We also tested whether a single vaccination could protect chickens from lethal disease, and in this context, we analyzed the level of sterile immunity in those immunized chickens. Finally, we present data showing the high stability of the H5 oligomer crude extracts after long-term storage.

## Materials and Methods

### Construction of plant expression vectors

**Fig. 1:**
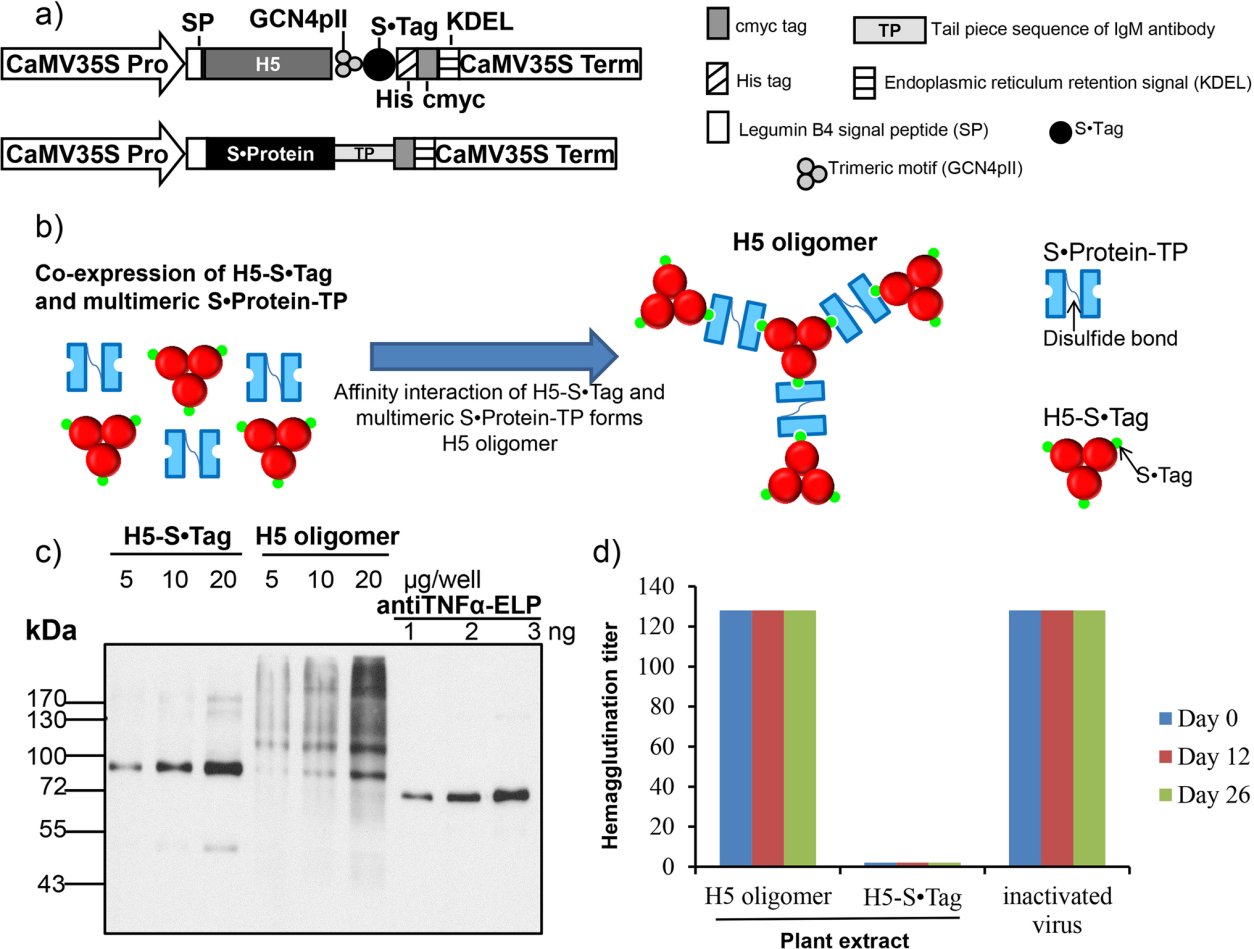
Analysis of H5 oligomers in plant crude extracts after transient expression. **a** Expression cassettes for the *in planta* production of H5-S•Tag trimers and S•Protein-TP protein. The ectodomain of hemagglutinin (H5) was trimerized by c-terminal fusion of the trimeric motif GCN4pII. The tailpiece (TP) element of mouse IgM was fused to S•Protein (S•Protein-TP). Recombinant H5 was fused to a c-myc tag to allow downstream detection by Western blot and a His-tag to facilitate their purification by IMAC. The legumine B4 signal peptide and the KDEL motif were used to allow retention in the endoplasmic reticulum. Pro: Cauliflower mosaic virus 35S ubiquitous promoter; Term: Cauliflower mosaic virus 35S terminator. **b** Schematic model of *in planta* formation of H5 oligomers via S•Tag::S•protein interaction and disulfide bridge formation. **c** H5 -S•Tag trimer and H5 oligomer antigen content analyzed by Western blot. 5, 10, and 20 µg whole plant proteins separated by SDS-PAGE. 1, 2, and 3 ng purified anti-TNFα-nanobody-ELP used as standards. Detection via anti-c-myc-antibodies, anti-mouse-IgG peroxidase, and ECL. **d** Stability analysis of H5-S•Tag trimers and H5 oligomers by hemagglutination titer estimation after 0, 12, and 26 days storage of plant crude extracts at 4 °C compared to the hemagglutination titer of stored inactivated rg A/swan/Germany/R65/ 2006(H5N1) viruses. Tests have been done in triplicate and all replications gave hemagglutination titers of 128 (SD = 0)

The DNA sequence encoding the ectodomain of H5 was obtained from the sequence of the H5N1 A/Chicken/DL/NAVET_0292/2013 strain. The H5 sequence was codon-optimized for expression in *Nicotiana benthamiana* (GeneBank accession number: SUB10611334 Optimized OL337990), artificially synthesized, and supplied in a cloning pUC57-H5 vector by GeneCust. The H5 fragment was amplified by using specific primers including *Bam*HI (agggatccGATCATATCTGCATCGGTTATCA) and *Psp*OMI (aggggcccTTCAAGTTTCACGCCAGAAATT) sites at the 5’ end. The PCR reaction was carried out as follows: denaturation at 94 °C for 3 min, 35 cycles of 94 °C for 30 s, 58 °C for 30 s, 72 °C for 1 min 30 s, elongation at 72 °C for 10 min, and cooling at 4 °C. The PCR product was separated on a 1% agarose gel, purified, and inserted into the pRTRA vector (Phan et al. 2017) containing trimeric motif (GCN4pII) and S•Tag at the sites of *Bam*HI and *Psp*OMI. The expression cassettes harboring sequences of Cauliflower mosaic virus (CaMV) 35S Promoter, Legumin B4 signal peptide (SP), the H5 fused with trimeric motif (GCN4pII) and S•Tag, a HIS-Tag, a c-myc-Tag, a KDEL motif for ER retention, and a terminator sequence (Fig. 1a) were inserted into the pCB301-Kana vector (Xiang et al. 1999) at the *Hin*dIII site. The recombinant pCB301-H5 S•Tag plasmid was then transformed into *Agrobacterium tumefaciens* pGV 2206 strain by electroporation. The *A. tumefaciens* harboring pCB301-S•Protein-TP was used as previously described (Phan et al. 2017).

### Plant Material

*N. benthamiana* plants (6–8 weeks old) were grown in a greenhouse at 55% humidity and a temperature of 22 °C. The periods for light and dark in the greenhouse were 16 h and 8 h, respectively.

### Agrobacterium infiltration

The Agrobacterium infiltration protocol was conducted as previously described (Phan et al. 2017). Briefly, to express H5-S•Tag protein in plants, the *A. tumefaciens* clones harboring pCB301-H5-S•Tag or pCB301-S•Protein-TP coding genes or a plant vector containing the HcPro gene that was coding for a silencing suppressor protein were cultured in LB medium containing 50 µg/mL kanamycin, 50 µg/mL carbenicillin and 50 µg/mL rifampicin for 2 days at 28 °C, and collected by centrifugation at 4000 rpm, 30 min at 4 °C. Cells from three bacterial strains were dissolved and diluted in an infiltration buffer [10 mM 2-(N-morpholino) ethanesulfonic acid (MES), 10 mM MgSO4, pH 5.6] to an OD_600_ between 0.6 and 1.0. To express a single H5-S•Tag protein in plants, the bacterial suspension of H5-S•Tag was mixed with the bacterial suspension of HcPro at a ratio of 1:1 (v/v). To facilitate the expression of H5 oligomers based on S•Tag::S•Protein interaction in planta, the mixture of bacterial suspensions of H5-S•Tag, S•Protein-TP and HcPro at a ratio of 1:1:1 (v/v) was prepared. For the generation of oligomers, 2 different agrobacterial clones are necessary: one for the production of S•Protein-TP (dimerized by disulfide bridges between TPs) and one for the production of H5-S•Tag trimers. The model of how H5 oligomers are formed is presented in Fig. 1b. The agrobacterial clone harboring HcPro was always used as a third clone or co-transformation to enhance the expression of H5-S•Tag trimers and H5 oligomers. *A. tumefaciens* suspensions were transiently infiltrated into *N. benthamiana* leaves by using the vacuum system for 1 min 30 s. After infiltration, the plants were kept in the greenhouse for six days in the same condition as above. The plant leaves were collected on day 6 and stored at -80 °C for further analysis.

### Plant protein extracts

Leaf samples were homogenized in liquid nitrogen before mixing with PBS buffer (137 mM NaCl, 2.7 mM KCl, 10 mM Na_2_HPO_4_, 1.8 mM KH_2_PO_4_, pH 7.4) at a ratio of 1:3 (w/v), respectively. To clarify the plant extract, centrifugation at 16 200 g for 30 min at 4 °C was conducted twice. The supernatant containing total soluble protein (TSP) was collected and called crude extract. The amount of TSP in plant extracts was determined via Bradford assay. Recombinant H5 contents in the crude plant were semi-quantified by Western blot.

Known amounts of the anti-TNFα-nanobody-ELP standard protein (Conrad et al. 2011) containing the c-myc -tag were used in the same membrane to construct blot signal intensities. Hemagglutinin amounts in the plant crude extracts were determined by comparing their blot signal intensities with blot signal intensities of standard proteins. Plant extracts were mixed with 50% glycerol and kept at -20 °C for later experiments.

### SDS-PAGE and Western blot analysis

The recombinant H5 proteins, S•Protein-TP and a standard protein (anti-TNFα-nanobody-ELP, Conrad et al. 2011) were separated in SDS-PAGE gel 10% at 100 V for 2 h. These proteins were then transferred to a Nitrocellulose membrane (Millipore) at 18 V overnight. The Western blot protocol was conducted as described by Gahrtz and Conrad (2009). Briefly, a solution containing 5% free-fat milk dissolved in TBS buffer was used to block the membrane for 2 h before incubation with monoclonal anti-c-myc antibodies. Briefly, a solution containing 5% free-fat milk dissolved in TBS buffer was used to block the membrane for 2 h before incubation with diluted home-made supernatant of monoclonal anti-c-myc antibodies (9E10) (1:50) for 2 h. The membrane was incubated with sheep anti-mouse IgG, horseradish peroxidase-linked whole antibody (GE Healthcare UK Limited, Little Chalfont Buckinghamshire, UK) as secondary antibodies for 1 h at room temperature. The signal was detected via enhanced chemiluminescence-based detection (ECL).

### Hemagglutinin purification by Immobilized metal affinity chromatography (IMAC)

H5 proteins from plants were purified by IMAC following the procedure as described previously (Phan et al. 2017). Briefly, the homogenized leaves were mixed with 50 mM Tris buffer (pH 8.0) at a ratio of 1:3 (w/v) in a blender. Centrifugation at 75 600 g, 4 °C for 30 min was applied before filtration by paper filters to clarify the plant extract. The clear plant extract was then combined with Ni–NTA agarose resin (Qiagen). The mixture was slightly stirred at 4 °C overnight and applied to a chromatography column. To remove non-bound protein, the column was loaded with washing buffer (50 mM NaH_2_PO_4_, 300 mM NaCl, 30 mM imidazole, pH 8.0). The purified H5 proteins were collected after the application of an elution buffer (50 mM NaH_2_PO_4_, 300 mM NaCl, 125 mM Imidazole, pH 8.0) onto the column. Finally, the purified H5 proteins were dialyzed in PBS buffer and concentrated by incubation of the dialysis bag in PEG6000. IMAC-purified proteins were subjected to size exclusion chromatography (SEC).

### SEC

SEC was performed following the protocol as described (Phan et al. 2017). Briefly, an equal amount of IMAC-purified H5-S•Tag trimer and H5 oligomers protein was applied onto a Superose™ 6 increase 10/300GL column (GE Healthcare). The molecular weight of H5 proteins was estimated via the application of a kit containing standard proteins with molecular weights from 44–2000 kDa (GE Healthcare) onto the column. All fractions were collected for further analysis via the Hemagglutination test and Western blot.

### Hemagglutination test and hemagglutination inhibition assay

The hemagglutination test and hemagglutination inhibition assay were conducted according to the guideline of OIE with three replicates for each sample. Briefly, with the hemagglutination assay, 50 µL of PBS were added firstly into all wells of a plastic V-bottom microtiter plate. Next, 50 µL of antigens were introduced in the first well of the plate, a total of 50 µl of two-fold serial dilution was performed on the entire rows. For the HA assay, inactivated rg A/swan/Germany/R65/2006(H5N1) was used. Next, 50 µL of 1% chicken red blood cells (RBCs) were placed into each well of the plate. The plate was finally incubated at 25 °C for 30 min. The results were visualized. Hemagglutination unit (HAU) was determined as the endpoint dilution properly agglutinating RBCs. For HI, inactivated virus H5/H5N1 strains clade 2.3.2.1c containing A/Chicken/DL/NAVET_0292/2013 and A/DK/VN/Bacninh/NCVD-17A384/2017 were used. Hemagglutination inhibition unit (HI titer) was defined as the endpoint dilution that inhibited the agglutination of RBCs.

### Chicken immunization, sample collection, and monitoring

Three-week-old chickens (NAVETCO, Vietnam) were prepared for immunization with plant crude extracts. Before vaccination, plant crude extracts containing H5 oligomers or H5-S•Tag trimers were prepared to obtain equal hemagglutinin content. After semi-quantification by Western blotting, the hemagglutinin antigen concentration in the plant extracts containing H5 oligomers or H5-S•Tag trimers was 0.534 ng/µl. The wild-type plant crude extracts or S•Protein-TP plant crude extracts were diluted to obtain the final TSP concentration of 2.5 µg/µl that was equal to the TSP in plant extracts containing H5 oligomers or H5-S•Tag trimers. All plant extracts were extensively mixed with in-house water in oil adjuvant (NAVETCO, Vietnam) at a ratio of 3:7. The adjuvanted mixtures were kept at 4 °C. The viral challenge experiments were conducted with two storage time points of the adjuvanted H5 oligomer plant crude extracts: 3.5 and 5.5 months. Adjuvanted PBS was included as a negative control group. Every chicken was vaccinated with 0.5 ml adjuvanted plant-based vaccine that contained 375 µg TSP. Each dose of adjuvanted the plant extracts containing H5 oligomer or H5-S•Tag trimer included 80 ng hemagglutinin antigens.

### Two dose chicken immunization

Each chicken (twelve chickens per group) were intramuscularly immunized with 375 µg TSP in plant crude extracts containing adjuvanted 80 ng H5 oligomers or 80 ng H5-S•Tag trimers at days 0 and 21. Two positive control groups (twelve chickens per group) were injected with one dose of the Navet-Vifluvac 2 (clade 2.3.2.1c strain) vaccine or Navet-Vifluvac 1 (clade 1 strain) vaccine according to the manufacturer’s recommendation. Three negative controls were received with adjuvanted wild-type plant crude extracts, adjuvanted S•Protein-TP plant crude extracts, and PBS (ten chickens per group). All chicken sera were collected for immune response analyses at day 34. One day later, all chickens were challenged with HPAIV A/Chicken/DL/NAVET_0292/2013 (H5N1) by a 10^6^ ELD50 dose. The chicken survival rate was recorded 10 days after the viral challenge. Chicken sera were collected before the first immunization and virus challenge, and after the 2^nd^ immunization. Chicken swabs were collected at days 38 and 45 to analyze the virus shedding by real-time RT-PCR.

### One dose chicken immunization

Each chicken (twelve chickens per group) were intramuscularly injected with 375 µg TSP in plant crude extracts containing adjuvanted 80 ng H5 oligomers or 375 µg TSP in plant crude extracts containing adjuvanted 80 ng H5S•Tag trimers at day 0. The positive control group (twelve chickens per group) was immunized with one dose of Navet-Vifluvac 2 (mixture of clade 2.3.2.1 strain (A/Hubei/1/2010(H5N1)-PR8-IDCDC-RG30) and clade 1: NIBRG-14 vaccine) according to the manufacturer’s recommendation. Two negative controls were vaccinated with adjuvanted PBS and adjuvanted WT plant crude extracts. Three weeks after the first immunization, all chicken sera were collected for immune response analyses. After serum collection, all chickens in five groups were challenged with HPAIV A/DK/VN/Bacninh/NCVD-17A384/2017 (H5N1) at a 10^6^ ELD50 dose per chicken. The chicken survival rate was recorded 10 days after the viral challenge. Chicken sera were also collected before the first immunization and after the virus challenge if the chickens survive. Sera were analyzed by the hemagglutination inhibition test and partially the IgY titer was measured. Chicken swabs were collected at the days of 38 and 45 to analyze the virus shedding by real-timeRT-PCR.

### Indirect IgY ELISA

SEC-purified trimeric H5 protein was diluted in phage PBS (100 mM NaCl, 32 mM Na_2_HPO_4_, 17 mM Na_2_HPO_4_, pH 7.2), then applied on microtiter plates, and incubated at 4 °C overnight (Nalgen Nunc International, Roskilde, Denmark). The plates were blocked with PBS buffer containing 3% (w/v) bovine serum albumin (BSA), 0.05% (v/v) Tween20 (PBS-T) for 2 h at 25 °C. Chicken sera were diluted in PBS buffer including 1% BSA, applied to the plates and incubated for 2 h. To remove unbound proteins, the plates were washed five times with PBS-Tween for 5 min. Anti-chicken IgY (whole molecule) conjugated alkaline phosphatase antibodies (GE Healthcare) were diluted 35 000 times in phage PBS-T buffer containing 1% BSA and used as a secondary antibody. The plates were incubated for 1 h at 25 °C after the introduction of the secondary antibody. p-Nitrophenyl phosphate (pNPP) was dissolved in 0.1 M diethanolamine-HCl (pH 9.8), and used as an enzyme–substrate applied to the plates. The plates were incubated at 37 °C for 1 h before measurement of the absorbance signal at 405 nm by ELISA reader. The IgY titer is presented as a ratio of dilution. We determined the cutoff value by an ELISA of dilutions of sera from chickens vaccinated with adjuvanted PBS (negative control group). An ELISA OD450nm value of 0.08 at 1:250 dilution was selected as the cutoff value. Serial dilutions of sera from all chickens vaccinated with H5 oligomer and H5-S•Tag trimer plant crude extracts and inactivated vaccines were analyzed. The dilution factors necessary to fit the cut-off value are designated as endpoint IgY titers.

### Determination of virus shedding via real-time reverse transcription polymerase chain reaction (RT-PCR)

The presence of wild-type H5N1 virus in the chicken swabs at 3 days, 10 days after co-localization, and 3 days, 10 days post-challenge were determined by real-time RT-PCR.Total RNAs were extracted from the chicken swab using an RNA extraction kit (Viral Gene-spin TM Viral DNA/RNA Extraction Kit, iNtRON) according to the manufacturer’s instruction. Next, RNA was subjected to a Onestep RT-PCR kit (Qiagen) with primers and probe targeting the H5 gene (Spackman et al. 2002). PCR reactions were conducted as follows: reverse transcription at 50 °C for 20 min, denaturation at 95 °C for 5 min, amplification for 45 cycles at 95 °C for 15 s and 60 °C for 30 s. Real-time RT-PCR was carried out on Applied Biosystems 7500 Fast Real-Time PCR (Thermo Fisher). Cycle threshold (Ct) was determined using the 7500 Fast software (Thermo Fisher). Positive samples were detected if Ct was ≤ 35. It was acceptable when the Ct of the positive control was equal to Ct ± 2 of control that was previously known, and the Ct of negative control was 0.

### Statistical analyses

A Mann–Whitney Rank-Sum test in Sigma Plot software was conducted to statistically analyze the HI data and chicken antibody titers. A significant difference was determined if p-values were less than 0.05.

## Results

### Recombinant hemagglutinin oligomers are produced *in planta*

The expression cassette hemagglutinin of H5-NAVA0292 from the virus strain HPAIV A/Chicken/DL/NAVET_0292/2013 (H5N1) (Table S1) has been selected for plant-based H5 oligomer production (Fig. 1a, b). After successful transient co-transformation of *N. benthamiana* plants with agrobacterial strains containing expression vectors with constructs coding for H5 S•Tag trimers and S•Protein-TP as well as after transformation with an agrobacterial strain containing expression vectors with constructs coding for H5 S•Tag trimers, plant crude extracts were analyzed by Western blot after separation in an SDS gel at denaturing conditions. In the case of trimers, a single band roughly reflecting the expected size of a monomer was visible (Fig. 1c). In the case of the oligomers, the denaturation by SDS and/or the cleavage of the disulfide bonds between the TP elements by ß-mercaptoethanol was not complete and several bands of higher molecular weight were visible. The amount of H5 oligomers or trimers in the crude extracts was semi-quantitatively estimated by comparison with different amounts of a standard protein with c-myc-Tag (anti-TNFa-ELP-c-myc, Conrad et al. 2011). The content of H5 oligomers or trimers in the crude extract was calculated to be approximately 1.6–2 mg per 1 kg fresh leaves.

The potential oligomers and the trimers were further characterized at native conditions to keep the oligomeric structure. H5-S•Tag trimers and H5 oligomers were purified by IMAC using the HIS-Tag and then separated by size exclusion chromatography according to their molecular weight (SEC). SEC fractions corresponding to H5 oligomers and purified H5-S•Tag trimers were collected. The hemagglutination titer and the presence of H5 by Western blot of every fraction were estimated. High hemagglutination titers were observed in fractions A3 to A11 of H5 oligomers. The highest molecular weight (fraction A3 and A4, approximately 2000 kDa) corresponds to the highest hemagglutination titer (128, Figure S1). The analysis of H5-S•Tag trimers by SEC did not show high molecular weights or high hemagglutination titers (Figure S1). Very high molecular weight hemagglutinins (> 400 kDa to 2000 kDa, fractions A11 to A3) were exclusively detected in H5 oligomer extracts after co-expression of H5-S•Tag trimer and S•Protein-TP. This result indicated that the H5 oligomers were a mixture of oligomers that are made from different numbers of H5-S•Tag and S•Protein-TP incorporated into the complexes. Based on the calculated size of H5-S•Tag trimers (200 kDa), and the size of H5 oligomers determined by SEC, the number of H5-S•Tag trimers integrated into H5 oligomers ranged from 1 to 8.

For further characterization the crude extracts containing H5 oligomers or H5-S•Tag trimers were directly compared to inactivated rg A/swan/Germany/R65/2006(H5N1) viruses by the hemagglutination assay. Very high hemagglutination titers comparable to titers of inactivated virus preparations were measured for oligomers (Fig. 1d). These high titers are a strong argument for large and stable oligomeric structures in crude extracts. These titers were stable after storage of the crude extracts at 4 °C for at least 26 days (Fig. 1d). This result is an important prerequisite for chicken immunization experiments because identical aliquots of an extract could be used for immunizations at different time points.

### Chicken immunization with H5 oligomers in plant crude extracts causes immunity against avian flu

**Fig. 2:**
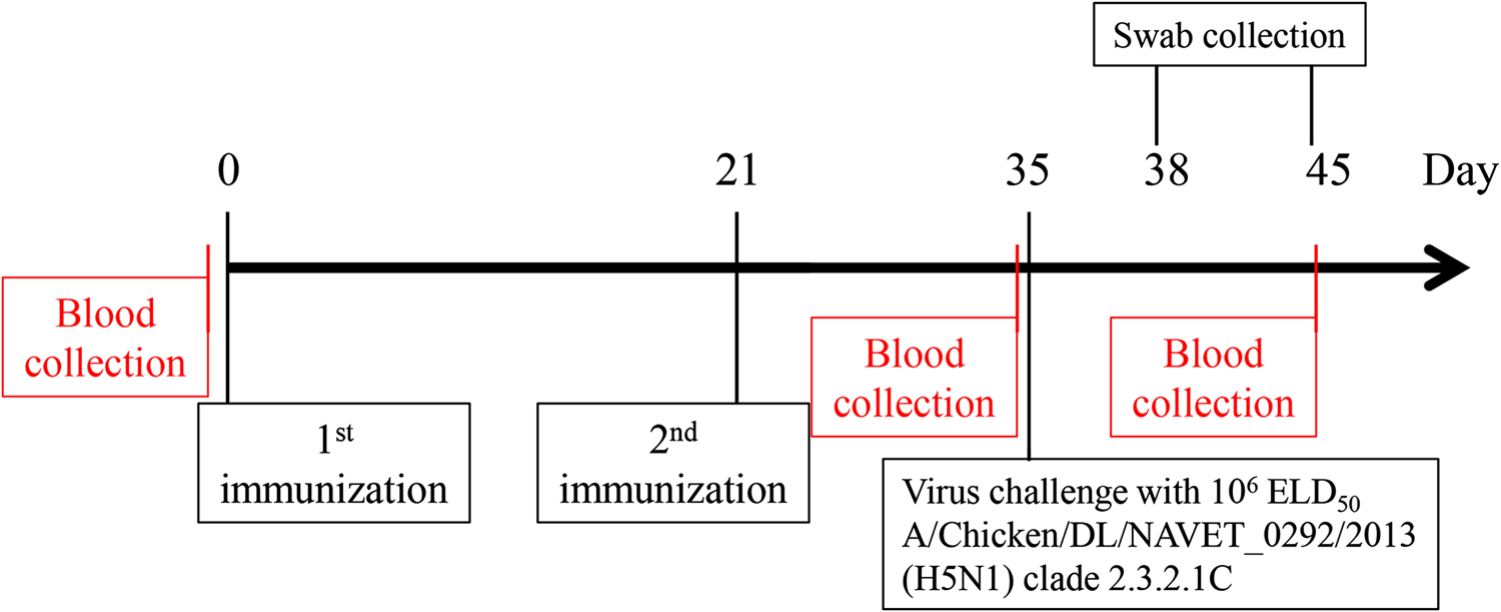
Chicken immunization, bleeding, and virus challenge schedules. Twelve chickens per group were intramuscularly immunized with adjuvanted H5 oligomer and H5- S•Tag trimer plant crude extracts at days 0 and 21. Twelve chickens in the positive control group received the Navet-Vifluvac 2 (clade 2.3.2.1c strain) vaccine. 12 chicken received Navet-Vifluvac 1 (clade 1 vaccine, A/Vietnam/1194/2004 (H5N1) NIBRG-14. Figure S1). Adjuvanted wildtype plant crude extracts, adjuvanted S•Protein-TP plant crude extracts, and PBS (10 chickens) were included as negative controls. Two weeks after the 2nd immunization, all chicken sera were collected for immune response analyses. Fourteen days after the 2nd immunization, all chickens were challenged with HPAIV A/Chicken/DL/NAVET_0292/2013 (H5N1) at a 10^6^ ELD50 dose. The chicken survival rate was recorded 10 days after the viral challenge. Chicken sera were collected before the first immunization and virus challenge, and after the 2nd immunization

The hemagglutinin content in the H5 oligomer and H5-S•Tag trimer plant crude extracts has been semi-quantitatively estimated by Western blot and frozen at -20 °C with 50% glycerol. The immunogenicity of plant extracts containing 80 ng H5 oligomers or 80 ng H5-S•Tag trimers (always 375 µg TSP, see Materials and Methods) has then been tested in chicken. A subsequent challenge experiment with HPAIV A/Chicken/DL/NAVET_0292/2013 (H5N1) virus was conducted to analyze the immunogenicity of plant-based vaccine candidates (Fig. 2).

**Fig. 3:**
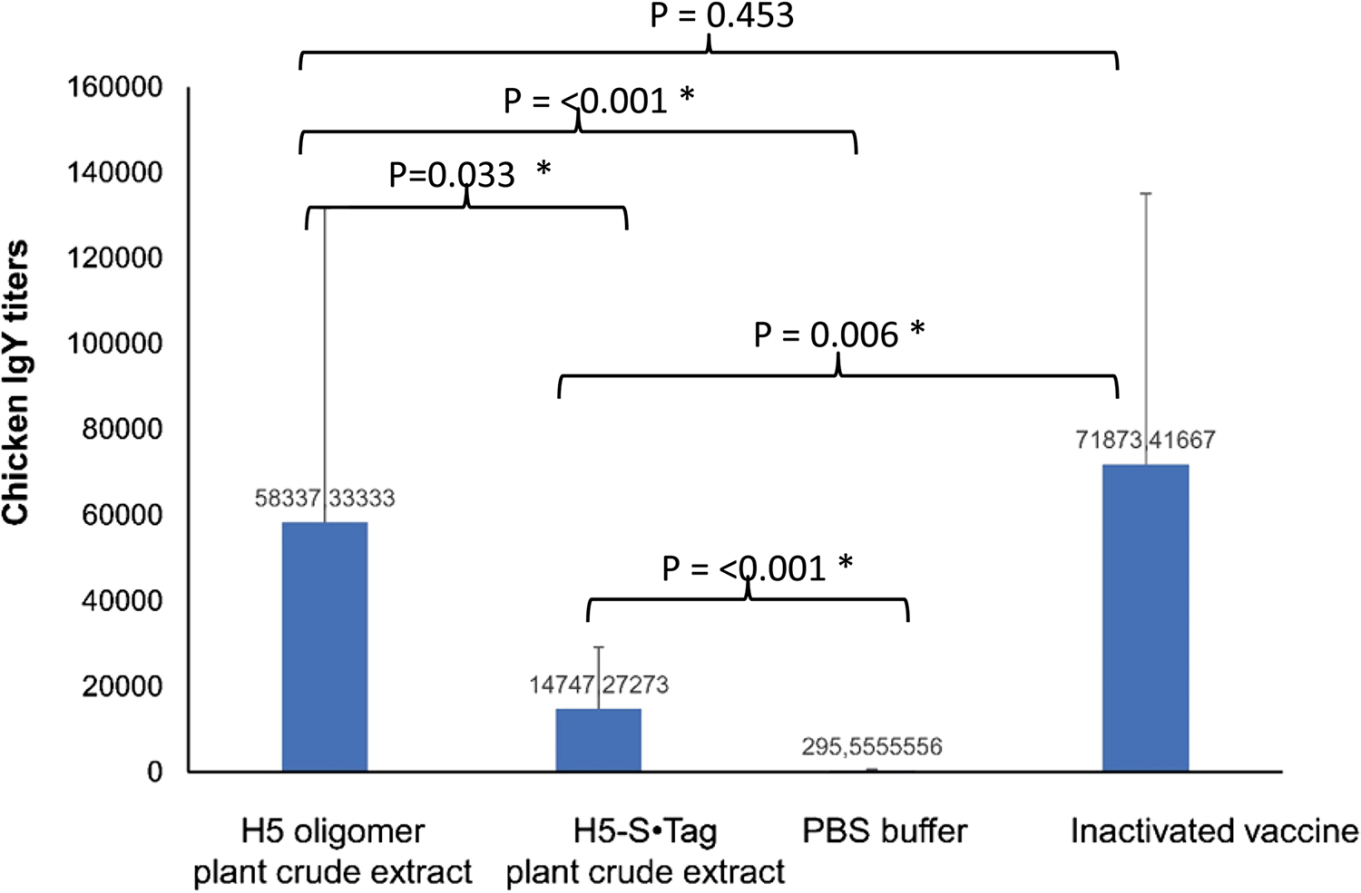
IgY titers of sera from vaccinated chickens. The endpoint IgY titers of chicken sera after vaccination with two doses of either adjuvanted H5 oligomer and adjuvanted H5-S•Tag trimer plant crude extracts, PBS, or inactivated vaccine (Navet-Vifluvac 2 NAVETCO). *: shows statistic difference. The titers are given as dilution factors necessary to fit the cut-off value

At first, we tested the humoral immune responses in the immunized chicken against the HPAIV A/Chicken/DL/NAVET_0292/2013 (H5N1) virus. For this purpose, we analyzed the endpoint IgY titers by an indirect ELISA (see Methods). As shown in Fig. 3, the chicken IgY responses elicited by the adjuvanted PBS had an endpoint IgY titer as low as 295.55. In contrast, the immunization with adjuvanted H5 oligomer and H5-S•Tag crude extracts and with the commercial inactivated H5N1 virus vaccine (Navet-Vifluvac 2) elicited strong IgY responses. Their endpoint IgY titers reached 58 337.33, 14 747.27, and 71 873.41, respectively. Notably, IgY immune responses induced by adjuvanted H5 oligomer crude extract were significantly better than those elicited by adjuvanted H5-S•Tag trimer crude extract (Fig. 3).

**Fig. 4:**
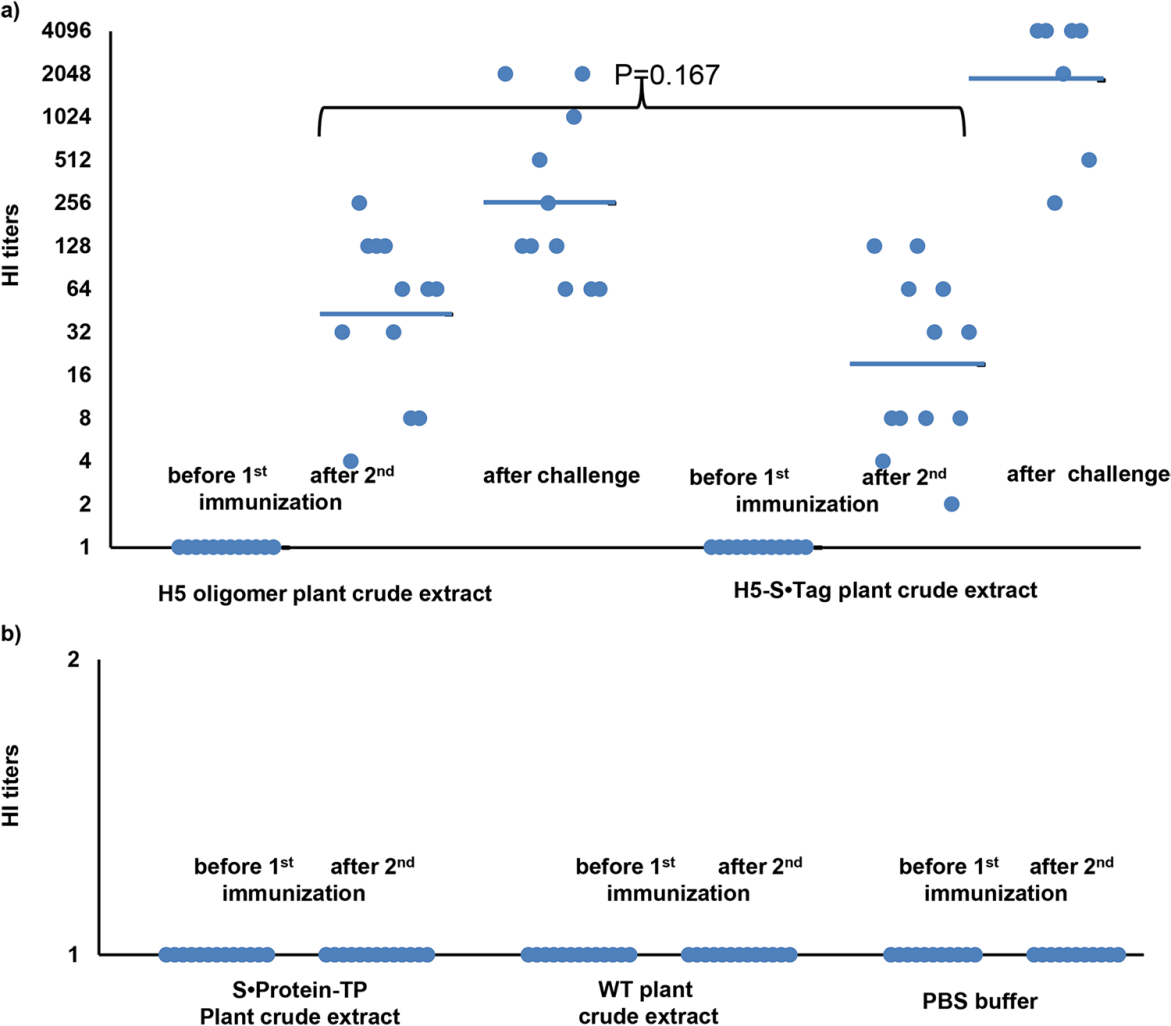
Chickens produce neutralizing antibodies after immunization with H5 oligomer plant crude extracts. **a** Immune responses after immunization with H5 oligomer and H5-S•Tag trimer plant crude extracts. Chicken antibody responses before the first immunization and after the second immunization with adjuvanted H5 oligomer and adjuvanted H5-S•Tag trimer plant crude extracts were measured by hemagglutination inhibition assay. Sera were also collected ten days after the HPAIV A/Chicken/DL/NAVET_0292/2013 (H5N1) challenge and measured by hemagglutination inhibition assay. A single dot represents the hemagglutination inhibition titer of a single serum sample. Bars are the geometric mean titers of each test group. **b** Immune responses after immunization with S•Protein-TP plant crude extract, wild type (WT) plant crude extracts, and PBS buffer. Chicken antibody responses before the first immunization and after the second immunization with adjuvanted S•Protein-TP crude extracts, adjuvanted wild type (WT) plant crude extracts, and PBS buffer were measured by hemagglutination inhibition assay. A single dot represents the hemagglutination inhibition titer of a single serum sample. Bars are the geometric mean titers of each test group

All chicken sera were tested by hemagglutination inhibition assays for the presence of neutralizing antibodies. For this purpose, inactivated viruses of the strain H5N1 2.3.2.1c/TTCD92 (clade 2.3.2.1c) were used. HI geometric mean titers (HI GMTs) of all sera from chickens before the first immunization with H5 oligomer plant crude extracts or H5-S•Tag trimer plant crude extracts showed no hemagglutination inhibition. At this time point, HI GMTs of sera from these two groups were 1 (Fig. 4a). The HI geometric mean titers of chicken sera from the S•Protein-TP, the wild type (WT) plant crude extracts, and the PBS buffer group were also 1 before the first immunization and stayed 1 after the second immunization. No neutralizing antibodies were induced in these chickens (Fig. 4b). Chicken vaccinated with the NAVETCO inactivated vaccine products (positive control groups), showed positive HI titers after the second immunization. Animals vaccinated with the Navet-Vifluvac 2 (clade 2.3.2.1c strain) had higher HI titers than chicken vaccinated with Navet-Vifluvac 1 (clade 1) (data not shown). This reflects the sequence differences between the two vaccine strains (91% similarity, Table S2). After the viral challenge, all sera from survived chickens from all groups were collected to measure neutralizing antibodies. Survived chicken sera showed higher HI titers compared to those before the viral challenge. At this time point, HI GMT titers of chicken immunized with H5 oligomer plant crude extracts or H5-S•Tag trimer plant crude extracts were 256 and 1855, respectively (Fig. 4a). This reflects that after the viral challenge, wild-type H5N1 viruses could replicate in vaccinated chickens. However, chickens vaccinated with H5 oligomers and H5-S•Tag trimer containing plant crude extracts established neutralizing antibodies before the viral challenge. These neutralizing antibodies could protect vaccinated chickens from lethal H5N1 virus infection.

**Fig. 5:**
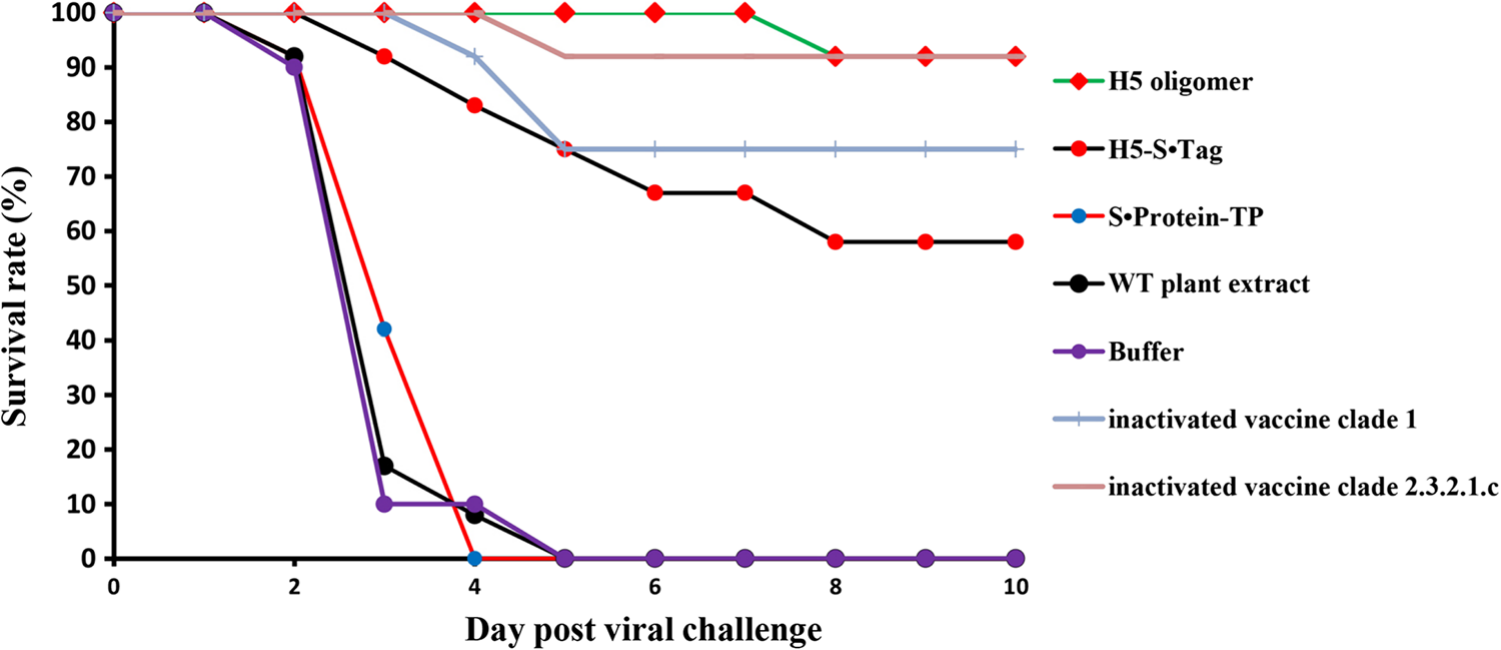
The chicken survival rate after immunization with plant-based vaccines. Twelve chickens per group were intramuscularly immunized with adjuvanted or non-adjuvanted H5 oligomer and H5-S•Tag trimer plant crude extracts at days 0 and 21. Adjuvanted WT plant crude extract (negative control) and inactivated virus of two different clades (positive controls, see legend Fig. 2) were included. Thirteen days after the 2nd immunization, all chicken sera were collected for immune response analyses. Fourteen days after the 2nd immunization, all chickens were challenged with HPAIV A/Chicken/DL/NAVET_0292/2013 (H5N1) at a 10^6^ ELD50 dose. The chicken survival rate was recorded 10 days after the viral challenge

The survival rate of chickens immunized with H5 oligomer plant crude extracts 10 days after challenge was 92% (11 of 12 animals, Fig. 5), whereas the survival rate of chickens immunized with H5 -S•Tag trimer plant crude extracts was 58% (7 of 12 animals, Fig. 5). The survival rate of chickens immunized with either Navet-Vifluvac 2 (clade 2.3.2.1c strain) vaccine or Navet-Vifluvac 1 (clade 1) vaccine (A/Vietnam/1194/2004 (H5N1) NIBRG-14, Table S1) were 92% (11 of 12 animals, Fig. 5) or 75% (9 of 12 animals, Fig. 5). This difference reflects the sequence differences between the two vaccine strains (see above, Table S2). These results show clearly, that adjuvanted H5 oligomer crude extracts are suitable H5N1 candidate vaccines. The data also show that H5 oligomer crude extracts protect chicken better from H5N1 viruses than H5-S•Tag crude extracts.

### One dose chicken immunization with H5 oligomers in plant crude extracts causes immunity against avian flu

**Fig. 6:**
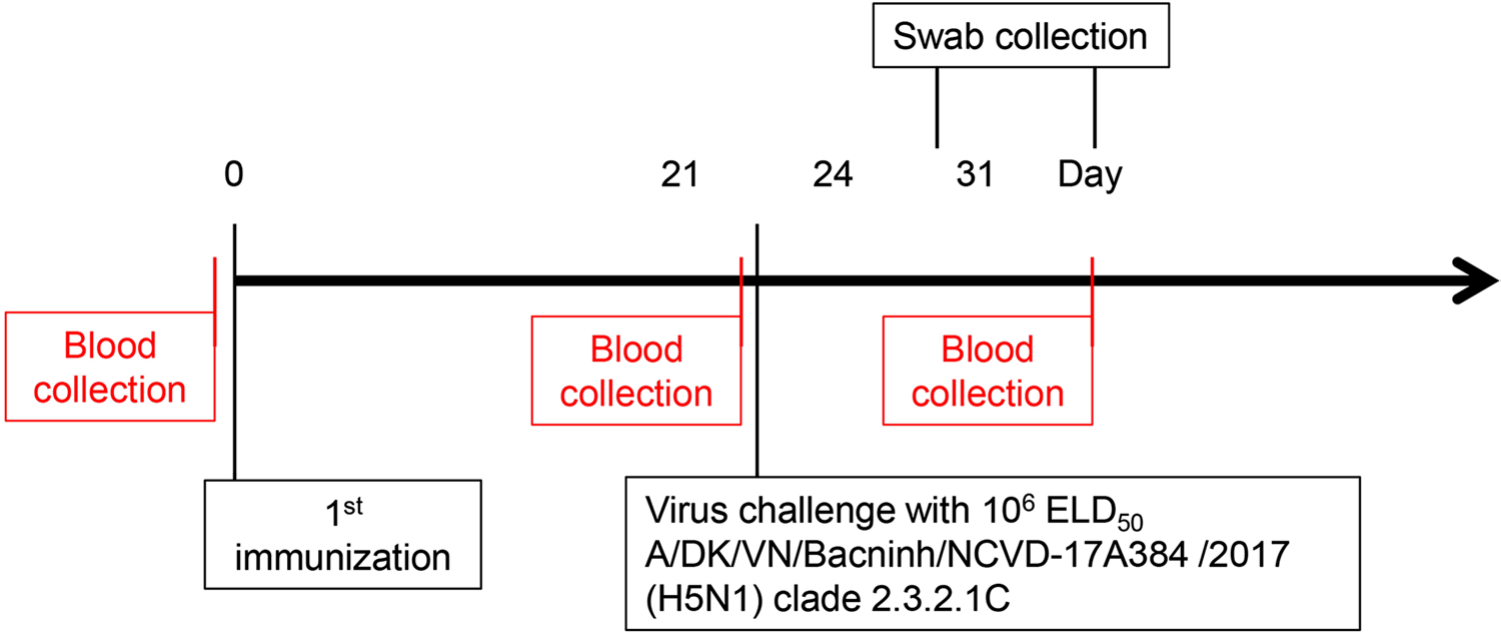
One dose chicken immunization, bleeding, and virus challenge schedules. Twelve chickens per group were intramuscular with adjuvanted H5 oligomer plant crude extract and H5-S•Tag trimer plant crude extracts immunized at day 0. Every 12 chickens were immunized with adjuvanted PBS, adjuvanted WT plant crude extracts (negative control groups), or Navet-Vifluvac 2 (mixture of clade 2.3.2.1 strain (A/Hubei/1/2010(H5N1)-PR8-IDCDC-RG30) and clade 1: NIBRG-14) vaccine (positive control group). Three weeks after the first immunization, all chicken sera were collected for immune response analyses. After serum collection, all chickens in five groups were challenged with HPAIV A/DK/VN/Bacninh/NCVD-17A384/2017 (H5N1) at a 10^6^ ELD50 dose per chicken. The chicken survival rate was recorded 10 days after the viral challenge. Chicken sera were also collected before the first immunization and after the virus challenge if chicken survive. Sera were analyzed by the hemagglutination inhibition test and partially the IgY titer was measured

One dose immunization would be a major economical and administrative benefit. To test this approach, one dose of either plant crude extracts (always 375 µg TSP) containing 80 ng H5 oligomers or, plant crude extracts containing 80 ng H5 -S•Tag trimers, wild-type plant crude extract (negative control), PBS (negative control), or inactivated vaccine (Navet – Fluvac 2 lot: 04.060618 (clade 2.3.2.1c strain)) were tested in chickens. Ten days after the wild-type H5 virus challenge sera were collected from survived chickens for HI measurement. The inactivated viruses of the strain H5N1 A/DK/VN/Bacninh/NCVD-17A384/2017 (clade 2.3.2.1c) were used for the HI assay. Swab samples were collected three and ten days after the virus challenge for analysis of virus presence by real-time RT-PCR (Fig. 6).

**Fig. 7:**
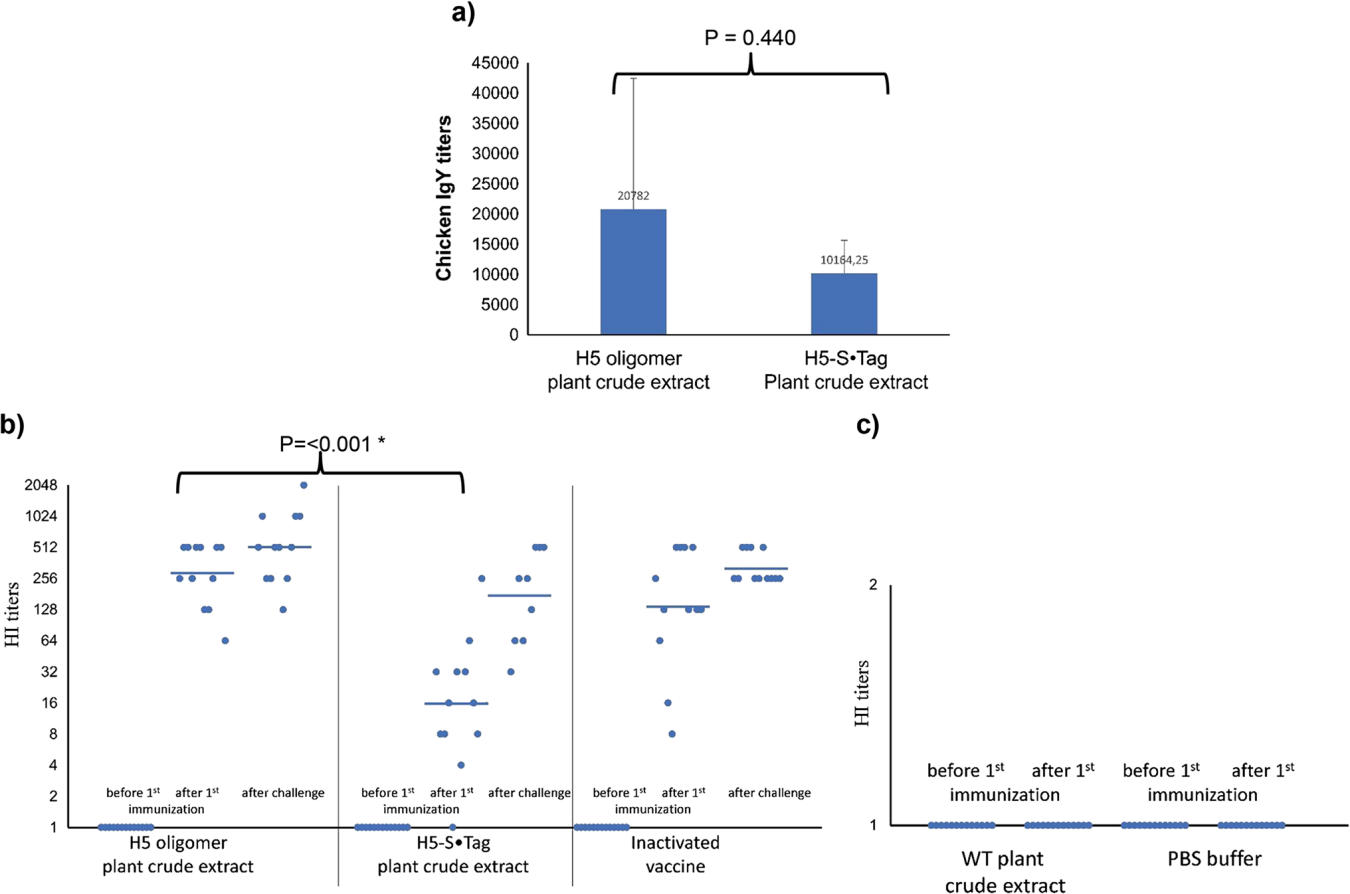
Neutralizing immune responses induced in chicken after one dose immunization with adjuvanted plant-based candidate vaccines. **a** IgY titers of sera from chickens vaccinated with one dose of H5 oligomer crude extract or one dose of H5-S•Tag trimer plant crude extract. **b** Chicken immune responses rose by one dose of H5 oligomer plant crude extracts, H5-S•Tag trimer plant crude extracts, and inactivated virus vaccine (Navet-Vifluvac 2) were analyzed by hemagglutination inhibition assay**.** *: shows statistic difference. **c** Chicken immune responses rose by PBS and wild-type (WT) plant crude extracts were analyzed by hemagglutination inhibition assay

**Fig. 8:**
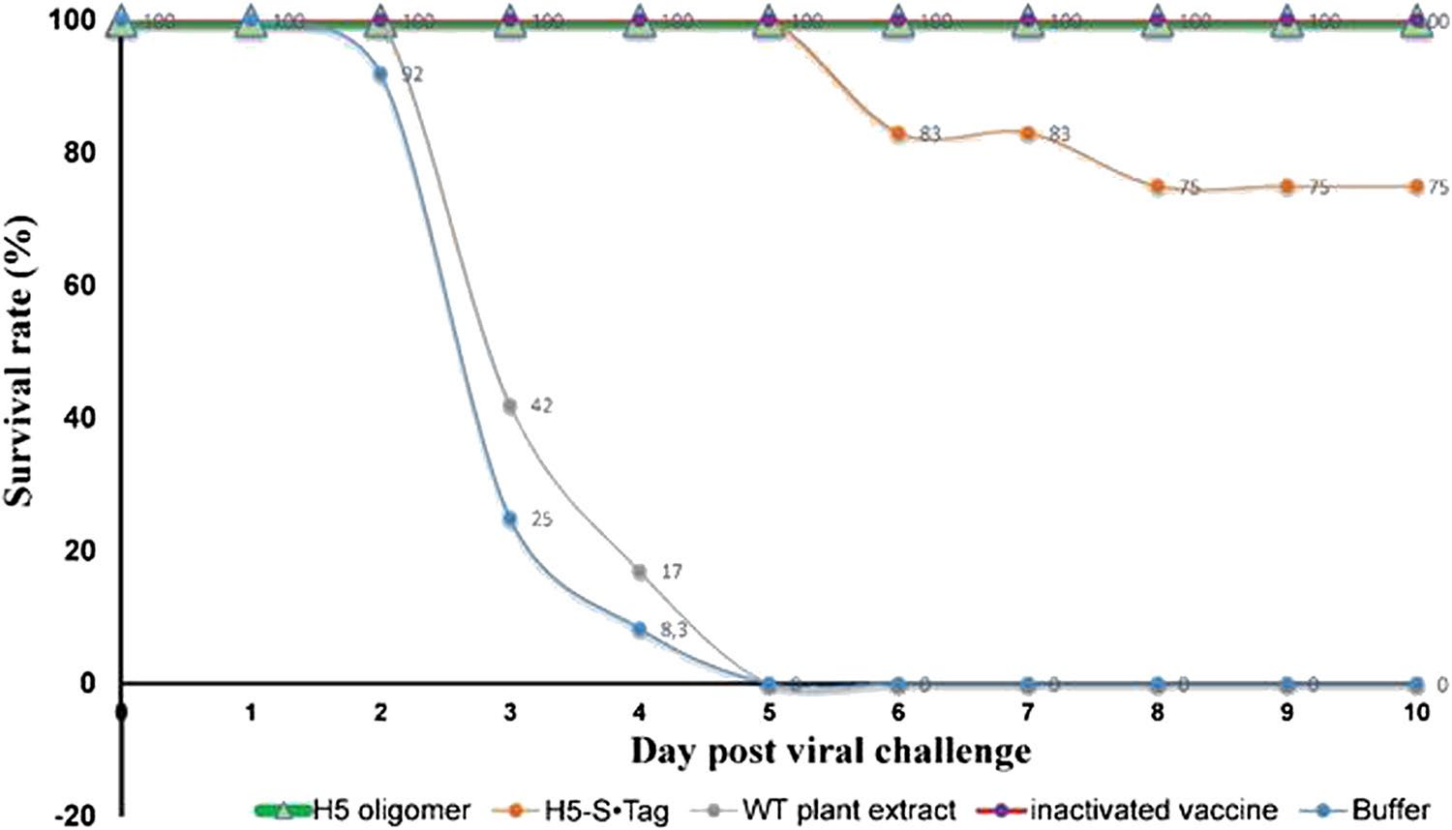
The chicken survival rate after one dose immunization with plant-based candidate vaccines. Twelve chickens per group were intramuscularly immunized with one dose of adjuvanted H5 oligomer plant crude extract or one dose H5-S•Tag trimer plant crude extracts at day 01. Immunization with adjuvanted WT plant crude extract and PBS as negative control groups and Navet-Vifluvac 2 as positive control group were included. Three weeks after the first immunization, all chicken sera were collected for immune response analyses by HI assay. Three weeks after the first immunization, all chickens were challenged with HPAIV A/DK/VN/Bacninh/NCVD-17A384/2017 (H5N1) at a 10^6^ ELD50 dose. The chicken survival rate was recorded 10 days after the viral challenge

Immune responses induced by adjuvanted H5 oligomer and H5-S•Tag trimer plant crude extracts after one dose immunization were analyzed by indirect ELISA. Their endpoint IgY titers were 20 782.00 and 10 164.25, respectively (Fig. 7a). As expected, H5 oligomer plant crude extracts induced stronger humoral immune responses than H5-S•Tag trimer plant crude extracts. All chicken sera were analyzed for the presence of neutralizing antibodies against inactivated H5 virus by hemagglutination inhibition tests. The HI geometric mean titers of all chicken sera were 1 before the first immunization. In the case of the negative control group sera (PBS and wild type (WT) plant crude extracts) no neutralizing antibodies could be detected after immunization (Fig. 7b). Three weeks after the first immunization, HI GMTs from chickens vaccinated with H5 oligomer and H5-S•Tag trimer plant crude extracts significantly increased. Chicken HI GMTs of these two groups were 287.4 and 16.0, respectively (Fig. 7c). This result indicated again that chicken immunized with H5 oligomer plant crude extracts show stronger neutralizing antibody responses than those immunized with H5-S•Tag trimer plant crude extracts. These neutralizing antibodies could protect vaccinated chickens from lethal H5N1 virus infection. The survival rate of chickens vaccinated with H5 oligomers plant crude extract was 100% (12 of 12 animals, Fig. 8, Table 1), while the chicken group vaccinated with H5-S•Tag trimer plant crude extract had 75% survivors (9 of 12 animals, Fig. 8, Table 1). Ten days after the viral challenge, sera from survived chickens showed higher HI titers compared to those before the viral challenge. At this time point, HI GMT titers of these groups were 512.2 and 174.2, respectively (Fig. 7a). After the viral challenge wild-type H5N1, viruses could induce more neutralizing antibodies. To get more inside into the resistance development, we analyzed the virus presence 3 and 10 days after the challenge in collected swabs by real-time RT-PCR (Table 1, Table S3). H5N1 viruses were detected in 58–75% of chickens vaccinated with adjuvanted-H5 oligomer, H5-S•Tag trimer plant crude extracts, or inactivated virus vaccines. In 75 to 100% of chickens from negative control groups, H5N1 viruses were also detected. Ten days after the lethal viral challenge, the presence of wild-type H5N1 viruses in swab samples was reduced, but still detected in 33, 44, and 17% of chickens vaccinated with adjuvanted-H5 oligomer, H5-S•Tag trimer plant crude extracts, and an inactivated virus vaccine, respectively (Table 1). This means we achieved a sterile immunity only in a part of the surviving chickens. These findings were further analyzed by a an experiment of housing vaccinated and non-vaccinated chicken together. All survived chickens (12 of 12 animals) vaccinated with H5 oligomer plant crude extract were transferred and co-localized with 10 non-vaccinated chickens negative in real-time RT-PCR (Table 2, Table S4). The chicken survival rate was recorded for further 10 days. All 22 chickens in this experiment survived. Three immunized chickens that survived the viral challenge contained H5N1 viruses 10 days after the challenge, but only one animal contained H5N1 viruses at day 10 of housing together with non-immunized chickens. In one animal of the non-vaccinated group (1 of 10) H5N1 viruses were detected after 10 days of housing together with infected vaccinated chickens. Although the infected chicken did not die, this result indicated that non-vaccinated chickens could in principle be infected because of partly non-sterile immunization. Taken together, the viral challenge in chicken showed that H5 oligomer containing plant crude extract could be used as an H5N1 candidate vaccine with only one dose immunization. Overall, the one-dose experiment and the two-dose experiment showed both high protection of chickens (n = 12 per group) vaccinated with plant crude extract containing H5 oligomer and H5-S•Tag trimer after virus challenge (survival rate > 90%). Immunization with one dose showed slightly better efficacy than immunization with two doses. This might be due to the limited number of birds (12) per group, not sufficient to ensure smaller differences.

**Table 1:** Analysis of wild type H5N1 virus presence three days and ten days after the viral challenge in collected swabs by real-time RT-PCR

Group	Real-time RT-PCR		
	3 days after the challenge	10 days after the challenge	Survival rate 10 days after the challenge
H5 oligomer plant crude extract	7/12: +	3/12: +	12/12
H5-S•Tag trimer plant crude extract	9/12: +	4/9: +	9/12
Wild type plant crude extract	9/12: +	NA	0/12
PBS	12/12: +	NA	0/12
Navet-Fluvac 2 vaccine (clade 2.3.2.1c)	7/12: +	2/12	12/12

( +): positive result, Ct value ≤ 35; (-): negative result, Ct value > 35. NA: not analyzed because chickens in PBS group died 10 days after challenge

*NA* not analyzed

**Table 2:** Housing of challenged vaccinated chickens together with non-immunized chickens

	No. chickens	HI GMT		RT-PCR	
		10 d.p.c	10 d.a.c-a	10 d.p.c	10 d.a.c-a
Survivals from H5 oligomer after the viral challenge	12	512.2	181.0	3/12	1/12
Non-vaccinated of co-localized chickens	10	1	1	0/10	1/10

( +): positive result, Ct value ≤ 35. 10 d.p.c: 10 days post challenge; 10 d.a.c-a: 10 days after co-localization

In the two experiments, we used different challenge viruses. In the challenge studies, we wanted to see, if our candidate vaccine could protect against H5N1 viruses in the clade 2.3.2.1c that are circulating in Vietnam. The two-dose experiment was performed with an HPAIV H5N1 virus clade 2.3.2.1c (A/Chicken/DL/NAVET0292/20132.3.2.1c/TTCD92) isolated in the southern region of Vietnam. The one-dose experiment was carried out with an HPAIV H5N1 virus clade 2.3.2.1c (A/DK/VN/Bacninh/NCVD-17A384/2017) isolated in the northern region of Vietnam. The use of different strains could also explain differences in efficacy. Finally, protection against two different virus strains from Vietnam could be shown.

### Immunogenicity of adjuvanted H5 oligomer plant crude extracts is maintained after long-time storage at moderate conditions

Long-time storage at rather moderate conditions is an important prerequisite for the successful development and application of a vaccine. The vaccine storage was assessed by a challenge experiment in chickens. H5 oligomer plant crude extracts were mixed with in-house water in oil adjuvant (NAVETCO) at a ratio of 3:7. The adjuvanted mixtures were kept at 4 °C. The viral challenge experiments were conducted with two storage time points of the adjuvanted H5 oligomer plant crude extracts: 3.5 and 5.5 months. Adjuvanted PBS was included as a negative control group (every ten chickens). Each of the twelve chickens was vaccinated with one dose of candidate vaccine stored at different times (see also Fig. 6). All chicken sera from negative controls and from chicken before the first vaccination showed negative hemagglutination inhibition results (HI GMTs 1, Table 3). Sera from chickens vaccinated with candidate vaccines stored 3.5 and 5.5 months showed HI GMTs as high as 157.6 and 294.1, respectively. Three weeks after the first vaccination all chickens were challenged with HPAIV A/DK/VN/Bacninh/NCVD-17A384/2017 (H5N1) at a 10^6^ ELD50 dose. Chicken survival was monitored for 10 days. All chickens from negative control groups died, while all chickens vaccinated with both stored adjuvanted H5 oligomer plant crude extracts survived. These data show that the capacity of adjuvanted H5 oligomer plant crude extracts to protect the chicken from lethal disease after an HPAIV H5N1 challenge was maintained even after 5.5 months incubation at 4 °C.

**Table 3:** Vaccine storage experiment

	Storage time	No. chickens	HI GMT		Survival rate
			Before vaccination	Three weeks after vaccination	
H5 oligomer plant extract	3.5 month	12	1	157.6	12/12
Negative group		10	1	1	0/10
H5 oligomer plant extract	5.5 month	12	1	294.1	12/12
Negative group		10	1	1	0/10

Plant crude extracts containing oligomeric hemagglutinins protect chickens against highly Pathogenic

## Discussion

Multivalent antigen presentation has been ascertained in different ways in vaccine development. Principally, multivalent antigens can be produced by using nanoparticles that display antigens (Kanekiyo et al. 2013) and result in either interior or exterior modifications to viral surfaces. Plant-virus-derived nanoparticles can support vaccine development (Steele et al. 2017).

The attachment of antigens to biodegradable polymers is a further option for vaccine development using multivalent antigens (Han et al. 2018). The production of trimeric forms of the ectodomain of the major antigen hemagglutinin has been proven as highly immunogenic for swine flu (H1N1) (Shoji et al. 2013) and avian flu (H5N1) (Phan et al. 2013). We also demonstrated the formation of oligomers based on Strep-Tactin®XT-Streptag®II interaction (Phan et al. 2018). The production of virus-like-particles from hemagglutinin is a further technology that has been applied for the production of human vaccines (D ´Aoust et al. 2008; Landry et al. 2010; D’Aoust et al. 2010). Multivalent antigen production platforms generate stronger immune responses because of superantigen presentation (Kanekiyo et al. 2013, 2015). All these vaccine production strategies need extended purification of components and in vitro assembly and/or purification of the end product. In some cases, they also require enrichment because of low concentrations in plant extracts. Downstream processing, including purification plant vaccines, is time-consuming and its cost can be calculated as much as 80% of the total costs for manufacturing (Kolotilin et al. 2014). Therefore, the reduction of cost in downstream processing of mass immunizations via the application of plant materials that are partially or minimally processed could be a key priority for the application of this technology in a veterinary environment (Wilken and Nikolov 2012). This is in contradiction with the cost-effectiveness needed for a production platform for veterinary vaccines. Therefore, we developed different oligomerization concepts which focus on forming oligomers in the endoplasmic reticulum of plant cells. Because of ER retention, hemagglutinins are not secreted and VLPs cannot be formed. We combined trimerization with the help of the GCN4pII motif with oligomerization by disulfide bonds formed by cysteine residues in the tailpiece sequence (TP) from the C-terminal sequence of IgM, by homoantiparallel peptide interaction (HAP) and homodimer protein interaction (HDP) (Phan et al. 2020). In a further approach, we produced stable and very large hemagglutinin H5 oligomers by the specific interaction between S•Tag and S•Protein. Trimerized hemagglutinins with S•Tag were combined with S•Protein-TP (Phan et al. 2017). In all four cases, the oligomers were formed *in planta* in the endoplasmic reticulum. Crude extracts also induced strong immune reactions in mice demonstrated by neutralizing antibody responses measured by HI tests (Phan et al. 2020, 2017). Chickens immunized with adjuvanted H5 oligomer-TP crude extracts were protected from lethal disease to a high percentage (Phan et al. 2020). In the actual paper, we show, that oligomerization by on S•Tag::S•Protein interaction was successful also with different H5 variants (Table S1), thus underlining the usefulness of this strategy. We also show, that the concentration of H5 oligomers combined via S•Tag::S•Protein interaction was sufficient to induce strong humoral immune responses, strong neutralizing antibody responses, and resistance after challenge with a wild type HPAIV H5 virus strain. In all three parameters, crude extracts with H5 oligomers induced better responses than crude extracts with trimers (Figs. 3, 4, 5). The results of the one-dose chicken immunization experiment are encouraging. The neutralizing antibodies induced by one immunization with adjuvanted crude extract protected vaccinated chickens from lethal H5N1 virus to 100% (Fig. 8, Table 1). The analysis of virus presence 3 and 10 days after the challenge in collected swabs shows that virus titers are 0 in some chickens but viruses were present in about one-third of chickens immunized with H5 oligomer crude extracts. In one of three chickens containing H5N1 viruses 10 days after challenge viruses were still present after 10 days of housing with non-vaccinated chickens and in only one of ten non-immunized chickens H5N1 viruses were detected after 10 days of co-localization. All chickens survived. The exact degree of immunity has to be further defined using more extended experiments and also using a higher number of chickens.

Stability analyses of H5 oligomer crude extracts have already been performed by hemagglutination tests and stability on ice for 41 days has been shown (Phan et al. 2020). The H5 oligomer crude extracts were mixed with adjuvants and stored for a more extended period. The stability was measured by the immunization of chickens and viral challenge experiments. High stability was indirectly shown by 100% survival of the chickens immunized with adjuvanted H5 oligomer crude extracts. This stability allows storage without freezing and combined with high immunogenicity it is a strong argument for the development of a vaccine that fits into the cost frame necessary for veterinary vaccines. However, there are limitations when using plant crude extracts from original *Nicotiana* species leaves. One important point to be taken into account is the content of nicotine and other alkaloids even in *Nicotiana benthamiana* (Moghbel et al. 2017). Extraction technologies have to be further developed to minimize the alkaloid content and more extended chicken treatments and veterinary analyses with such extracts have to be performed to avoid regulatory problems. After transient expression, endotoxins from *Agrobacteria* cannot be excluded per se and related analyses including veterinary analyses of chickens have to be performed. Furthermore, transient expression *in planta* has to be developed as a safe industrial process, that guarantees comparable expression levels.

The data we present here show that the H5 oligomer crude extracts induce strong virus-neutralizing immune responses even after only one dose immunization comparable to commercial vaccines. The cost-effective production of plant crude extract vaccine candidates and the high stability after long-time storage will force the further exploration of this technology for veterinary vaccine development.

## Supplementary Information

Below is the link to the electronic supplementary material.

Supplementary file1 (DOCX 24 kb) Table S1. Comparison of amino acid H5 sequences of 2 H5N1 strains and nucleotide sequence of constructs used in this study

Supplementary file2 (DOCX 14 kb) Table S2. Sequence pair distances

Supplementary file3 (DOCX 20 kb) Table S3. Analysis of wild-type H5N1 virus presence three days and ten days after the viral challenge in collected swabs by real-time RT-PCR

Supplementary file4 (DOCX 15 kb) Table S4. Analysis of wild-type H5N1 virus presence after housing together experiments after by real-time RT-PCR

Supplementary file5 (JPG 660 kb) Figure S1. Formation of oligomeric H5

## Data Availability

The sequence data used in this study are given in Materials and Methods and Supplementary Material. All other datasets generated during and/or analysed during the current study are available from the corresponding author on reasonable request.

## References

[CR1] Andre FE (2003). Vaccinology: past achievements, present roadblocks and future promises. Vaccine.

[CR2] Backer MV, Aloise R, Przekop K, Stoletov K, Backer JM (2002). Molecular Vehicles for Targeted Drug Delivery. Bioconjugate Chem.

[CR3] Chen Q, Lai H, Hurtado J, Stahnke J, Leuzinger K, Dent M (2013). Agroiniltration as an efective and scalable strategy of gene delivery for production of pharmaceutical proteins. Adv Tech Biol Med.

[CR4] Conrad U, Plagmann I, Malchow S, Sack M, Floss DM, Kruglov AA (2011). ELPylated anti-human TNF therapeutic single-domain antibodies for prevention of lethal septic shock. Plant Biotechnol J.

[CR5] Cox RJ, Brokstad KA, Ogra P (2004). Influenza virus: immunity and vaccination strategies comparison of the immune response to inactivated and live, attenuated influenza vaccines. Scand J Immunol.

[CR6] D’Aoust MA, Lavoie PO, Couture MM, Trépanier S, Guay JM, Dargis M (2008). Influenza virus-like particles produced by transient expression in *Nicotiana benthamiana* induce a protective immune response against a lethal viral challenge in mice. Plant Biotechnol J.

[CR7] D’Aoust MA, Couture MM, Charland N, Trépanier S, Landry N, Ors F (2010). The production of hemagglutinin-based virus-like particles in plants: a rapid, efficient and safe response to pandemic influenza. Plant Biotechnol J.

[CR8] European Centre for Disease Prevention and Control (ECDPC) (2020) Outbreak of highly pathogenic avian inluenza A(H5N8) in Europe. https://www.ecdc.europa.eu/en/publications-data/avian-influenza-overview-update-19-november-2020-eueea-and-uk. Accessed 05 Nov 2021

[CR9] Gahrtz M, Conrad U (2009). Immunomodulation of plant function by in vitro selected single-chain Fv intrabodies. Methods Mol Biol.

[CR10] Han J, Zhao D, Li D, Wang X, Jin Z, Zhao K (2018). Polymer-Based Nanomaterials and Applications for Vaccines and Drugs. Polymers (basel).

[CR11] Kanekiyo M, Bu W, Joyce MG, Meng G, Whittle JR, Baxa U (2015). Rational Design of an Epstein-Barr Virus Vaccine Targeting the Receptor-Binding Site. Cell1.

[CR12] Kanekiyo M, Wei CJ, Yassine HM, McTamney PM, Boyington JC, Whittle JR (2013). Self-assembling influenza nanoparticle vaccines elicit broadly neutralizing H1N1 antibodies. Nature.

[CR13] Kolotilin I, Topp E, Cox E, Devriendt B, Conrad U, Joensuu J (2014). Plant-based solutions for veterinary immunotherapeutics and prophylactics. Vet Res.

[CR14] Landry N, Ward BJ, Trépanier S, Montomoli E, Dargis M, Lapini G (2010). Preclinical and clinical development of plant-made virus-like particle vaccine against avian H5N1 influenza. PLoS ONE.

[CR15] Moghbel N, Ryu B, Ratsch A, Steadman KJ (2017). Nicotine alkaloid levels, and nicotine to nornicotine conversion, in Australian Nicotiana species used as chewing tobacco. Heliyon.

[CR16] Phan HT, Pohl J, Floss DM, Rabenstein F, Veits J, Le BT (2013). ELPylated haemagglutinins produced in tobacco plants induce potentially neutralizing antibodies against H5N1 viruses in mice. Plant Biotechnol J.

[CR17] Phan HT, Gresch U, Conrad U (2018). In vitro-Formulated Oligomers of Strep-Tagged Avian Influenza Haemagglutinin Produced in Plants Cause Neutralizing Immune Responses. Front Bioeng Biotechnol.

[CR18] Phan HT, Ho TT, Chu HH, Vu TH, Gresch U, Conrad U (2017). Neutralizing immune responses induced by oligomeric H5N1-hemagglutinins from plants. Vet Res.

[CR19] Phan HT, Pham VT, Ho TT, Pham NB, Chu HH, Vu TH (2020). Immunization with plant-derived multimeric H5 hemagglutinins protect chicken against highly pathogenic avian influenza virus H5N1. Vaccines.

[CR20] Raines RT, McCormick M, Van Oosbree TR, Mierendorf RC (2000). The S·tag fusion system for protein purification. Methods Enzymol.

[CR21] Shoji Y, Jones RM, Mett V, Chichester JA, Musiychuk K, Sun X (2013). A plant-produced H1N1 trimeric hemagglutinin protects mice from a lethal influenza virus challenge. Hum Vaccin Immunother.

[CR22] Steele JFC, Peyret H, Saunders K, Castells-Graells R, Marsian J, Meshcheriakova Y (2017). Synthetic plant virology for nanobiotechnology and nanomedicine. Wiley Interdiscip Rev Nanomed Nanobiotechnol.

[CR23] Spackman E, Senne DA, Myers TJ, Bulaga LL, Garber LP, Perdue ML (2002). Development of a real-time reverse transcriptase PCR assay for type A influenza virus and the avian H5 and H7 hemagglutinin subtypes. J Clin Microbiol.

[CR24] Takeyama N, Kiyono H, Yuki Y (2015) Plant-based vaccines for animals and humans: recent advances in technology and clinical trials. Ther Adv Vaccines:139–154. 10.1177/2051013615613272. 10.1177/2051013615613272 PMC4667769 26668752

[CR25] Topp E, Irwin R, McAllister T, Lessard M, Joensuu JJ, Kolotilin I (2016). The case for plant-made veterinary immunotherapeutics. Biotechnol Adv.

[CR26] Woolhouse ME, Gowtage-Sequeria S (2005). Host range and emerging and reemerging pathogens. Emerg Infect Dis.

[CR27] Wilken LR, Nikolov ZL (2012). Recovery and purification of plant-made recombinant proteins. Biotechnol Adv.

[CR28] Xiang C, Han P, Lutziger I, Wang K, Oliver DJ (1999). A mini binary vector series for plant transformation. Plant Mol Biol.

[CR29] Yen HL, Webster RG, Compans RW, Orenstein WA (2009). Pandemic influenza as a current threat vaccines for pandemic influenza. vaccines for pandemic influenza.

