# Supplementary material for: Plant crude extracts containing oligomeric hemagglutinins protect chickens against highly Pathogenic Avian Influenza Virus after one dose of immunization
Source: Vet Res Commun. 2022 May 28;47(1):191–205. doi: 10.1007/s11259-022-09942-3 (PMC9145123; doi:10.1007/s11259-022-09942-3)
Supplement: Supplementary file 1 — Supplementary file1 (DOCX 24 kb) Table S1. Comparison of amino acid H5 sequences of 2 H5N1 strains and nucleotide sequence of constructs used in this study [file 11259_2022_9942_MOESM1_ESM.docx]

**Table S1. Comparison of amino acid H5 sequences of 2 H5N1 strains and nucleotide sequences of plant expression vectors used in the actual study.**

H5-NAV0292: hemagglutinin from A/Chicken-DL-NAVET_0292-2013(H5N1) strain.

H5-17A384: hemagglutinin from A/DK/VN/Bacninh/NCVD-17A384/2017 strain. The H5 sequence from this strain was used to prove oligomeric formation based on S•Tag and S•Protein interaction in plants. Both A/Chicken-DL-NAVET_0292-2013(H5N1) and A/DK/VN/Bacninh/NCVD-17A384/2017 were classified into the clade 2.3.2.1c.

....|....| ....|....| ....|....| ....|....| ....|....| ....|....|

10 20 30 40 50 60

**H5-17A384**  SDHICIGYHA NNSTEQVDTI MEKNVTVTHA QDILEKTHNG KLCDLNGVKP LILKDCSVAG

**NIBRG-14**  ..Q....... .......... .......... .......... .....D.... ...R......

**H5-A/Hubei** .......... .......... .......... .......... .......... ..........

**H5-NAV0292** .......... .......... K......... .......R.. ........R. ......R.S.

....|....| ....|....| ....|....| ....|....| ....|....| ....|....|

70 80 90 100 110 120

**H5-17A384**  WLLGNPLCDE FTNVPEWSYI VEKANPANDL CYPGNFNDYE ELKHLLSRIN HFEKIQIIPK

**NIBRG-14**  ......M... .I........ ......V... ....D..... .......... ..........

**H5-A/Hubei** ......M... .I........ .......... .......... .......... ..........

**H5-NAV0292** .......... .SI....... .......... .......... .......... ........T.

....|....| ....|....| ....|....| ....|....| ....|....| ....|....|

130 140 150 160 170 180

**H5-17A384**  DSWSDHEASL GVSAACSYQG NSSFFRNVVW LIKKDNAYPT IKKGYNNTNR EDLLILWGIH

**NIBRG-14**  S...S..... ...S..P... K......... ....NST... ..RS.....Q ....V.....

**H5-A/Hubei** N......... ......P... K......... .......... .........Q ....V.....

**H5-NAV0292** .......... .......... .......... .......... .......... ..........

....|....| ....|....| ....|....| ....|....| ....|....| ....|....|

190 200 210 220 230 240

**H5-17A384**  HPNDEAEQTR LYQNPTTYIS IGTSTLNQRL VPKIATRSKI NGQSGRIDFF WTILKPNDAI

**NIBRG-14**  ....A....K .......... V......... ..R......V ......ME.. ..........

**H5-A/Hubei** .......... .......... .......... .......... .......... ..........

**H5-NAV0292** .......... .......... .......... .......... .......... ..........

....|....| ....|....| ....|....| ....|....| ....|....| ....|....|

250 260 270 280 290 300

**H5-17A384**  HFESNGNFIA PEYAYKIVKK GDSTIMRSEV EYGNCNTRCQ TPIGAINSSM PFHNIHPLTI

**NIBRG-14**  N......... .......... ......K..L .......K.. ..M....... ..........

**H5-A/Hubei** .......... .......... ......K... .......... .......... ..........

**H5-NAV0292** .......... .......... .......... .......... .......... ..........

....|....| ....|....| ....|....| ....|....| ....|....| ....|....|

310 320 330 340 350 360

**H5-17A384**  GECPKYVKSN KLVLATGLRN SPQRERRRKR GLFGAIAGFI EGGWQGMVDG WYGYHHSNEQ

**NIBRG-14**  .......... R......... .......... .......... .......... ..........

**H5-A/Hubei** .......... .......... .......... .......... .......... ..........

**H5-NAV0292** .......... .......... .......... .......... .......... ..........

....|....| ....|....| ....|....| ....|....| ....|....| ....|....|

370 380 390 400 410 420

**H5-17A384**  GSGYAADKES TQKAIDGVTN KVNSIIDKMN TQFEAVGREF NNLERRIENL NKKMEDGFLD

**NIBRG-14**  .......... .......... .......... .......... .......... ..........

**H5-A/Hubei** .......... .......... .......... .......... .......... ..........

**H5-NAV0292** .......... .......... .......... .......... .......... ..........

....|....| ....|....| ....|....| ....|....| ....|....| ....|....|

430 440 450 460 470 480

**H5-17A384**  VWTYNAELLV LMENERTLDF HDSNVKNLYD KVRLQLKDNA KELGNGCFEF YHKCNNECME

**NIBRG-14**  .......... .......... .......... ......R... .......... ....D.....

**H5-A/Hubei** .......... .......... .....R.... .......... .......... ....D.....

**H5-NAV0292** .......... .......... .......... .......... .......... ..........

....|....| ....|....| ....|....| ....|....| ....|....| ....|....|

490 500 510 520 530 540

**H5-17A384**  SVRNGTYDYP QYSEEARLKK EEISGVKLES IGIYQILSIY STVASSLVLA IMMAGLSLWM

**NIBRG-14**  .......... .........R .......... .......... .......A.. ..V.......

**H5-A/Hubei** .......... .........R .......... .......... .......... ..........

**H5-NAV0292** .......... .........R .......... .......... .......... ..........

.

**H5-17A384**  C

**NIBRG-14**  .

**H5-A/Hubei** .

**H5-NAV0292** .

**Plant expression vector pCB301-H5-S Tag**

CGCTCACCGGGCTGGTTGCCCTCGCCGCTGGGCTGGCGGCCGTCTATGGCCCTGAAACGCGCCAGAAACGCCGTCGAAGCCGTGTGCGAGACACCGCGGCCGCCGGCGTTGTGGATACCTCGCGGAAAACTTGGCCCTCACTGACAGATGAGGGGCGGACGTTGACACTTGAGGGGCCGACTCACCCGGCGCGGCGTTGACAGATGAGGGGCAGGCTCGATTTCGGCCGGCGACGTGGAGCTGGCCAGCCTCGCAAATCGGCGAAAACGCCTGATTTTACGCGAGTTTCCCACAGATGATGTGGACAAGCCTGGGGATAAGTGCCCTGCGGTATTGACACTTGAGGGGCGCGACTACTGACAGATGAGGGGCGCGATCCTTGACACTTGAGGGGCAGAGTGCTGACAGATGAGGGGCGCACCTATTGACATTTGAGGGGCTGTCCACAGGCAGAAAATCCAGCATTTGCAAGGGTTTCCGCCCGTTTTTCGGCCACCGCTAACCTGTCTTTTAACCTGCTTTTAAACCAATATTTATAAACCTTGTTTTTAACCAGGGCTGCGCCCTGTGCGCGTGACCGCGCACGCCGAAGGGGGGTGCCCCCCCTTCTCGAACCCTCCCGGCCCGCTCTCGAGTTGGCAGCATCACCCATAATTGTGGTTTCAAAATCGGCTCCGTCGATACTATGTTATACGCCAACTTTGAAAACAACTTTGAAAAAGCTGTTTTCTGGTATTTAAGGTTTTAGAATGCAAGGAACAGTGAATTGGAGTTCGTCTTGTTATAATTAGCTTCTTGGGGTATCTTTAAATACTGTAGAAAAGAGGAAGGAAATAATAAATGGCTAAAATGAGAATATCACCGGAATTGAAAAAACTGATCGAAAAATACCGCTGCGTAAAAGATACGGAAGGAATGTCTCCTGCTAAGGTATATAAGCTGGTGGGAGAAAATGAAAACCTATATTTAAAAATGACGGACAGCCGGTATAAAGGGACCACCTATGATGTGGAACGGGAAAAGGACATGATGCTATGGCTGGAAGGAAAGCTGCCTGTTCCAAAGGTCCTGCACTTTGAACGGCATGATGGCTGGAGCAATCTGCTCATGAGTGAGGCCGATGGCGTCCTTTGCTCGGAAGAGTATGAAGATGAACAAAGCCCTGAAAAGATTATCGAGCTGTATGCGGAGTGCATCAGGCTCTTTCACTCCATCGACATATCGGATTGTCCCTATACGAATAGCTTAGACAGCCGCTTAGCCGAATTGGATTACTTACTGAATAACGATCTGGCCGATGTGGATTGCGAAAACTGGGAAGAAGACACTCCATTTAAAGATCCGCGCGAGCTGTATGATTTTTTAAAGACGGAAAAGCCCGAAGAGGAACTTGTCTTTTCCCACGGCGACCTGGGAGACAGCAACATCTTTGTGAAAGATGGCAAAGTAAGTGGCTTTATTGATCTTGGGAGAAGCGGCAGGGCGGACAAGTGGTATGACATTGCCTTCTGCGTCCGGTCGATCAGGGAGGATATCGGGGAAGAACAGTATGTCGAGCTATTTTTTGACTTACTGGGGATCAAGCCTGATTGGGAGAAAATAAAATATTATATTTTACTGGATGAATTGTTTTAGTACCTAGATGTGGCGCAACGATGCCGGCGACAAGCAGGAGCGCACCGACTTCTTCCGCATCAAGTGTTTTGGCTCTCAGGCCGAGGCCCACGGCAAGTATTTGGGCAAGGGGTCGCTGGTATTCGTGCAGGGCAAGATTCGGAATACCAAGTACGAGAAGGACGGCCAGACGGTCTACGGGACCGACTTCATTGCCGATAAGGTGGATTATCTGGACACCAAGGCACCAGGCGGGTCAAATCAGGAATAAGGGCACATTGCCCCGGCGTGAGTCGGGGCAATCCCGCAAGGAGGGTGAATGAATCGGACGTTTGACCGGAAGGCATACAGGCAAGAACTGATCGACGCGGGGTTTTCCGCCGAGGATGCCGAAACCATCGCAAGCCGCACCGTCATGCGTGCGCCCCGCGAAACCTTCCAGTCCGTCGGCTCGATGGTCCAGCAAGCTACGGCCAAGATCGAGCGCGACAGCGTGCAACTGGCTCCCCCTGCCCTGCCCGCGCCATCGGCCGCCGTGGAGCGTTCGCGTCGTCTCGAACAGGAGGCGGCAGGTTTGGCGAAGTCGATGACCATCGACACGCGAGGAACTATGACGACCAAGAAGCGAAAAACCGCCGGCGAGGACCTGGCAAAACAGGTCAGCGAGGCCAAGCAGGCCGCGTTGCTGAAACACACGAAGCAGCAGATCAAGGAAATGCAGCTTTCCTTGTTCGATATTGCGCCGTGGCCGGACACGATGCGAGCGATGCCAAACGACACGGCCCGCTCTGCCCTGTTCACCACGCGCAACAAGAAAATCCCGCGCGAGGCGCTGCAAAACAAGGTCATTTTCCACGTCAACAAGGACGTGAAGATCACCTACACCGGCGTCGAGCTGCGGGCCGACGATGACGAACTGGTGTGGCAGCAGGTGTTGGAGTACGCGAAGCGCACCCCTATCGGCGAGCCGATCACCTTCACGTTCTACGAGCTTTGCCAGGACCTGGGCTGGTCGATCAATGGCCGGTATTACACGAAGGCCGAGGAATGCCTGTCGCGCCTACAGGCGACGGCGATGGGCTTCACGTCCGACCGCGTTGGGCACCTGGAATCGGTGTCGCTGCTGCACCGCTTCCGCGTCCTGGACCGTGGCAAGAAAACGTCCCGTTGCCAGGTCCTGATCGACGAGGAAATCGTCGTGCTGTTTGCTGGCGACCACTACACGAAATTCATATGGGAGAAGTACCGCAAGCTGTCGCCGACGGCCCGACGGATGTTCGACTATTTCAGCTCGCACCGGGAGCCGTACCCGCTCAAGCTGGAAACCTTCCGCCTCATGTGCGGATCGGATTCCACCCGCGTGAAGAAGTGGCGCGAGCAGGTCGGCGAAGCCTGCGAAGAGTTGCGAGGCAGCGGCCTGGTGGAACACGCCTGGGTCAATGATGACCTGGTGCATTGCAAACGCTAGGGCCTTGTGGGGTCAGTTCCGGCTGGGGGTTCAGCCAGCGCTTTACTGAGATCTGGGGAACCCTGTGGTTGGCATGCACATACAAATGGACGAACGGATAAACCTTTTCACGCCCTTTTAAATATCCGATTATTCTAATAAACGCTCTTTTCTCTTAGGTTTACCCGCCAATATATCCTGTCAAACACTGATAGTTTAAACTGAAGGCGGGAAACGACAATCTGATCATGAGCGGAGAATTAAGGGAGTCACGTTATGACCCCCGCCGATGACGCGGGACAAGCCGTTTTACGTTTGGAACTGACAGAACCGCAACGTTGAAGGAGCCACTCAGCCGCGGGTTTCTGGAGTTTAATGAGCTAAGCACATACGTCAGAAACCATTATTGCGCGTTCAAAAGTCGCCTAAGGTCACTATCAGCTAGCAAATATTTCTTGTCAAAAATGCTCCACTGACGTTCCATAAATTCCCCTCGGTATCCAATTAGAGTCTCATATTCACTCTCAATCCAAATAATCTGCACCGGATCTGGATCGTTTCGCATGATTGAACAAGATGGATTGCACGCAGGTTCTCCGGCCGCTTGGGTGGAGAGGCTATTCGGCTATGACTGGGCACAACAGACAATCGGCTGCTCTGATGCCGCCGTGTTCCGGCTGTCAGCGCAGGGGCGCCCGGTTCTTTTTGTCAAGACCGACCTGTCCGGTGCCCTGAATGAACTGCAGGACGAGGCAGCGCGGCTATCGTGGCTGGCCACGACGGGCGTTCCTTGCGCAGCTGTGCTCGACGTTGTCACTGAAGCGGGAAGGGACTGGCTGCTATTGGGCGAAGTGCCGGGGCAGGATCTCCTGTCATCTCACCTTGCTCCTGCCGAGAAAGTATCCATCATGGCTGATGCAATGCGGCGGCTGCATACGCTTGATCCGGCTACCTGCCCATTCGACCACCAAGCGAAACATCGCATCGAGCGAGCACGTACTCGGATGGAAGCCGGTCTTGTCGATCAGGATGATCTGGACGAAGAGCATCAGGGGCTCGCGCCAGCCGAACTGTTCGCCAGGCTCAAGGCGCGCATGCCCGACGGCGATGATCTCGTCGTGACCCATGGCGATGCCTGCTTGCCGAATATCATGGTGGAAAATGGCCGCTTTTCTGGATTCATCGACTGTGGCCGGCTGGGTGTGGCGGACCGCTATCAGGACATAGCGTTGGCTACCCGTGATATTGCTGAAGAGCTTGGCGGCGAATGGGCTGACCGCTTCCTCGTGCTTTACGGTATCGCCGCTCCCGATTCGCAGCGCATCGCCTTCTATCGCCTTCTTGACGAGTTCTTCTGAGCGGGACTCTGGGGTTCGAAATGACCGACCAAGCGACGCCCAACCTGCCATCACGAGATTTCGATTCCACCGCCGCCTTCTATGAAAGGTTGGGCTTCGGAATCGTTTTCCGGGACGCCGGCTGGATGATCCTCCAGCGCGGGGATCTCATGCTGGAGTTCTTCGCCCACGGGATCTCTGCGGAACAGGCGGTCGAAGGTGCCGATATCATTACGACAGCAACGGCCGACAAGCACAACGCCACGATCCTGAGCGACAATATGATCGGGCCCGGCGTCCACATCAACGGCGTCGGCGGCGACTGCCCAGGCAAGACCGAGATGCACCGCGATATCTTGCTGCGTTCGGATATTTTCGTGGAGTTCCCGCCACAGACCCGGATGATCCCCGATCGTTCAAACATTTGGCAATAAAGTTTCTTAAGATTGAATCCTGTTGCCGGTCTTGCGATGATTATCATATAATTTCTGTTGAATTACGTTAAGCATGTAATAATTAACATGTAATGCATGACGTTATTTATGAGATGGGTTTTTATGATTAGAGTCCCGCAATTATACATTTAATACGCGATAGAAAACAAAATATAGCGCGCAAACTAGGATAAATTATCGCGCGCGGTGTCATCTATGTTACTAGATCGGGCCAATACGCAAACCGCCTCTCCCCGCGCGTTGGCCGATTCATTAATGCAGCTGGCACGACAGGTTTCCCGACTGGAAAGCGGGCAGTGAGCGCAACGCAATTAATGTGAGTTAGCTCACTCATTAGGCACCCCAGGCTTTACACTTTATGCTTCCGGCTCGTATGTTGTGTGGAATTGTGAGCGGATAACAATTTCACACAGGAAACAGCTATGACCATGATTACGCCAAGCTTGCATGCCTGCAGGTCACTGGATTTTGGTTTTAGGAATTAGAAATTTTATTGATAGAAGTATTTTACAAATACAAATACATACTAAGGGTTTCTTATATGCTCAACACATGAGCGAAACCCTATAAGAACCCTAATTCCCTTATCTGGGAACTACTCACACATTATTCTGGAGAAAATAGAGAGAGATAGATTTGTAGAGAGAGACTGGTGATTTTTGCGGACTCTATCGACGGATCGGGCTAGAGTTCGTCTTTGGAACCATTCAGATCCTCTTCTGAGATGAGTTTTTGTTCTGCGGCCaaATGGTGATGGTGGTGATGCgcggccgcGGAATCCATATGCTGTCTTTCGAACTTAGCTGCTGCTGTCTCTTTgctagcTGAACCACCTCCACCTCTTTCTCCAATAAGTTTCTTGATACGAGCGATCTCGTTCTCGATATGATAAATCTTGGAAAGAATTTCCTCGATCTTATCCTCGATCTGCTTCATTCTCTTgggcccTTCAAGTTTCACGCCAGAAATTTCTTCCCTTTTGAGCCTGGCTTCTTCAGAATACTGCGGATAATCATAAGTCCCATTCCTCACTGATTCCATGCATTCATTATTGCATTTGTGATAAAATTCAAAACACCCATTCCCGAGTTCTTTGGCATTATCTTTGAGCTGGAGCCTCACTTTGTCATAGAGATTTTTCACATTAGAATCGTGAAAATCAAGAGTACGCTCATTTTCCATAAGGACCAAAAGCTCAGCATTATAAGTCCACACATCAAGAAAGCCGTCCTCCATCTTCTTGTTCAAATTTTCAATACGACGTTCAAGATTATTGAATTCACGCCCGACAGCCTCAAACTGAGTATTCATTTTATCAATAATACTATTGACTTTGTTAGTCACGCCATCAATAGCTTTCTGAGTAGATTCTTTATCAGCAGCATAACCACTACCCTGTTCATTAGAATGATGGTAACCATACCAACCATCAACCATTCCCTGCCATCCACCTTCAATGAAACCAGCAATTGCTCCAAAAAGACCCCTTCTTTCTCGTTGGGGAGAATTTCGCAATCCAGTAGCCAGAACCAGTTTGTTAGATTTCACGTATTTGGGACACTCACCAATGGTGAGAGGGTGAATATTATGAAAAGGCATGCTGCTATTAATTGCTCCTATTGGGGTTTGACACCTGGTATTACAATTTCCGTATTCAACCTCGCTTCTCATTATGGTGCTATCCCCTTTCTTGACAATCTTGTATGCGTACTCCGGAGCGATAAAATTACCATTGCTCTCAAAGTGTATTGCGTCATTAGGTTTCAAAATGGTCCAAAAGAAGTCTATTCTTCCGGATTGACCGTTTATCTTAGATCTCGTTGCTATCTTTGGAACCAGTCTCTGATTAAGAGTAGACGTGCCGATGCTAATGTATGTTGTAGGATTcTGGTAAAGTCTAGTTTGTTCTGCCTCGTCATTTGGATGGTGAATTCCCCATAAAATCAGAAGATCCTCCCGATTGGTGTTGTTGTAACCCTTCTTAATTGTTGGGTATGCGTTGTCCTTCTTAATCAACCAAACAACGTTTCGAAAGAATGAAGAGTTACCTTGATAACTACAAGCAGCACTAACTCCTAAAGATGCCTCATGGTcTGACCAGGAGTCCTTCGGAATAATTTGGATCTTCTCGAAATGGTTGATTCTGGATAACAGATGCTTTAACTCCTCGTAATCGTTGAAATTTCCAGGGTAACACAAATCATTAGCAGGGTTTGcCTTCTCAACGATATAACTCCACTCTGGAACGTTTgTGAATTCATCACATAATGGATTACCTAGCAACCATCCAGCAACGGAACAATCCTTTAGGATCAAAGGCTTAACTCCGTTTAAGTCGCACAACTTTCCGTTATGTGTCTTCTCTAGGATATCTTGTGCGTGTGTAACTGTTACGTTCTTTTCCATGATCGTATCTACTTGCTCTGTACTGTTGTTAGCATGATAACCGATGCAGATATGATCGGATCCTGCTAAACATGTGCTTGTAAAGAGAAGCAAGGAAAGTGAAAGCAAAGATAGAAAAGGTTTGGAAGCCATGGTTCCCGGCCGGGTCAGATCCTCTAGAGTCGATCGAGGTCCTCTCCAAATGAAATGAACTTCCTTATATAGAGGAAGGGTCTTGCGAAGGATAGTGGGATTGTGCGTCATCCCTTACGTCAGTGGAGATATCACATCAATCCACTTGCTTTGAAGACGTGGTTGGAACGTCTTCTTTTTCCACGATGTTCCTCGTGGGTGGGGGTCCATCTTTGGGACCACTGTCGGTAGAGGCATTCTTGAACGATAGCCTTTCCTTTATCGCAATGATGGCATTTGTAGAAGCCATCTTCCTTTTCTACTGTCCTTTCGATGAAGTGACAGATAGCTGGGCAATGGAATCCGAGGAGGTTTCCCGATATTACCCTTTGTTGAAAAGTCTCAATAGCCCTCTGGTCTTCTGAGACTGTATCTTTGATATTCTTGGAGTAGACGAGAGTGTCGTGCTCCACCATGTTGACCTGCAGGCATGCAAGCTTATCGATACCGTCGACCTCGAGGGGGGGCCCGGTACCAAAACCACCCCAGTACATTAAAAACGTCCGCAATGTGTTATTAAGTTGTCTAAGCGTCAATTTGTTTACACCACAATATATCCTGCCACCAGCCAGCCAACAGCTCCCCGACCGGCAGCTCGGCACAAAATCACCACTCGATACAGGCAGCCCATCAGTCCACTAGA

**Plant expression vector pCB301-S Protein-TP**

CGCTCACCGGGCTGGTTGCCCTCGCCGCTGGGCTGGCGGCCGTCTATGGCCCTGCAAACGCGCCAGAAACGCCGTCGAAGCCGTGTGCGAGACACCGCGGCCGCCGGCGTTGTGGATACCTCGCGGAAAACTTGGCCCTCACTGACAGATGAGGGGCGGACGTTGACACTTGAGGGGCCGACTCACCCGGCGCGGCGTTGACAGATGAGGGGCAGGCTCGATTTCGGCCGGCGACGTGGAGCTGGCCAGCCTCGCAAATCGGCGAAAACGCCTGATTTTACGCGAGTTTCCCACAGATGATGTGGACAAGCCTGGGGATAAGTGCCCTGCGGTATTGACACTTGAGGGGCGCGACTACTGACAGATGAGGGGCGCGATCCTTGACACTTGAGGGGCAGAGTGCTGACAGATGAGGGGCGCACCTATTGACATTTGAGGGGCTGTCCACAGGCAGAAAATCCAGCATTTGCAAGGGTTTCCGCCCGTTTTTCGGCCACCGCTAACCTGTCTTTTAACCTGCTTTTAAACCAATATTTATAAACCTTGTTTTTAACCAGGGCTGCGCCCTGTGCGCGTGACCGCGCACGCCGAAGGGGGGTGCCCCCCCTTCTCGAACCCTCCCGGCCCGCTCTCGAGTTGGCAGCATCACCCATAATTGTGGTTTCAAAATCGGCTCCGTCGATACTATGTTATACGCCAACTTTGAAAACAACTTTGAAAAAGCTGTTTTCTGGTATTTAAGGTTTTAGAATGCAAGGAACAGTGAATTGGAGTTCGTCTTGTTATAATTAGCTTCTTGGGGTATCTTTAAATACTGTAGAAAAGAGGAAGGAAATAATAAATGGCTAAAATGAGAATATCACCGGAATTGAAAAAACTGATCGAAAAATACCGCTGCGTAAAAGATACGGAAGGAATGTCTCCTGCTAAGGTATATAAGCTGGTGGGAGAAAATGAAAACCTATATTTAAAAATGACGGACAGCCGGTATAAAGGGACCACCTATGATGTGGAACGGGAAAAGGACATGATGCTATGGCTGGAAGGAAAGCTGCCTGTTCCAAAGGTCCTGCACTTTGAACGGCATGATGGCTGGAGCAATCTGCTCATGAGTGAGGCCGATGGCGTCCTTTGCTCGGAAGAGTATGAAGATGAACAAAGCCCTGAAAAGATTATCGAGCTGTATGCGGAGTGCATCAGGCTCTTTCACTCCATCGACATATCGGATTGTCCCTATACGAATAGCTTAGACAGCCGCTTAGCCGAATTGGATTACTTACTGAATAACGATCTGGCCGATGTGGATTGCGAAAACTGGGAAGAAGACACTCCATTTAAAGATCCGCGCGAGCTGTATGATTTTTTAAAGACGGAAAAGCCCGAAGAGGAACTTGTCTTTTCCCACGGCGACCTGGGAGACAGCAACATCTTTGTGAAAGATGGCAAAGTAAGTGGCTTTATTGATCTTGGGAGAAGCGGCAGGGCGGACAAGTGGTATGACATTGCCTTCTGCGTCCGGTCGATCAGGGAGGATATCGGGGAAGAACAGTATGTCGAGCTATTTTTTGACTTACTGGGGATCAAGCCTGATTGGGAGAAAATAAAATATTATATTTTACTGGATGAATTGTTTTAGTACCTAGATGTGGCGCAACGATGCCGGCGACAAGCAGGAGCGCACCGACTTCTTCCGCATCAAGTGTTTTGGCTCTCAGGCCGAGGCCCACGGCAAGTATTTGGGCAAGGGGTCGCTGGTATTCGTGCAGGGCAAGATTCGGAATACCAAGTACGAGAAGGACGGCCAGACGGTCTACGGGACCGACTTCATTGCCGATAAGGTGGATTATCTGGACACCAAGGCACCAGGCGGGTCAAATCAGGAATAAGGGCACATTGCCCCGGCGTGAGTCGGGGCAATCCCGCAAGGAGGGTGAATGAATCGGACGTTTGACCGGAAGGCATACAGGCAAGAACTGATCGACGCGGGGTTTTCCGCCGAGGATGCCGAAACCATCGCAAGCCGCACCGTCATGCGTGCGCCCCGCGAAACCTTCCAGTCCGTCGGCTCGATGGTCCAGCAAGCTACGGCCAAGATCGAGCGCGACAGCGTGCAACTGGCTCCCCCTGCCCTGCCCGCGCCATCGGCCGCCGTGGAGCGTTCGCGTCGTCTCGAACAGGAGGCGGCAGGTTTGGCGAAGTCGATGACCATCGACACGCGAGGAACTATGACGACCAAGAAGCGAAAAACCGCCGGCGAGGACCTGGCAAAACAGGTCAGCGAGGCCAAGCAGGCCGCGTTGCTGAAACACACGAAGCAGCAGATCAAGGAAATGCAGCTTTCCTTGTTCGATATTGCGCCGTGGCCGGACACGATGCGAGCGATGCCAAACGACACGGCCCGCTCTGCCCTGTTCACCACGCGCAACAAGAAAATCCCGCGCGAGGCGCTGCAAAACAAGGTCATTTTCCACGTCAACAAGGACGTGAAGATCACCTACACCGGCGTCGAGCTGCGGGCCGACGATGACGAACTGGTGTGGCAGCAGGTGTTGGAGTACGCGAAGCGCACCCCTATCGGCGAGCCGATCACCTTCACGTTCTACGAGCTTTGCCAGGACCTGGGCTGGTCGATCAATGGCCGGTATTACACGAAGGCCGAGGAATGCCTGTCGCGCCTACAGGCGACGGCGATGGGCTTCACGTCCGACCGCGTTGGGCACCTGGAATCGGTGTCGCTGCTGCACCGCTTCCGCGTCCTGGACCGTGGCAAGAAAACGTCCCGTTGCCAGGTCCTGATCGACGAGGAAATCGTCGTGCTGTTTGCTGGCGACCACTACACGAAATTCATATGGGAGAAGTACCGCAAGCTGTCGCCGACGGCCCGACGGATGTTCGACTATTTCAGCTCGCACCGGGAGCCGTACCCGCTCAAGCTGGAAACCTTCCGCCTCATGTGCGGATCGGATTCCACCCGCGTGAAGAAGTGGCGCGAGCAGGTCGGCGAAGCCTGCGAAGAGTTGCGAGGCAGCGGCCTGGTGGAACACGCCTGGGTCAATGATGACCTGGTGCATTGCAAACGCTAGGGCCTTGTGGGGTCAGTTCCGGCTGGGGGTTCAGCCAGCGCTTTACTGAGATCTGGGGAACCCTGTGGTTGGCATGCACATACAAATGGACGAACGGATAAACCTTTTCACGCCCTTTTAAATATCCGATTATTCTAATAAACGCTCTTTTCTCTTAGGTTTACCCGCCAATATATCCTGTCAAACACTGATAGTTTAAACTGAAGGCGGGAAACGACAATCTGATCATGAGCGGAGAATTAAGGGAGTCACGTTATGACCCCCGCCGATGACGCGGGACAAGCCGTTTTACGTTTGGAACTGACAGAACCGCAACGTTGAAGGAGCCACTCAGCCGCGGGTTTCTGGAGTTTAATGAGCTAAGCACATACGTCAGAAACCATTATTGCGCGTTCAAAAGTCGCCTAAGGTCACTATCAGCTAGCAAATATTTCTTGTCAAAAATGCTCCACTGACGTTCCATAAATTCCCCTCGGTATCCAATTAGAGTCTCATATTCACTCTCAATCCAAATAATCTGCACCGGATCTGGATCGTTTCGCATGATTGAACAAGATGGATTGCACGCAGGTTCTCCGGCCGCTTGGGTGGAGAGGCTATTCGGCTATGACTGGGCACAACAGACAATCGGCTGCTCTGATGCCGCCGTGTTCCGGCTGTCAGCGCAGGGGCGCCCGGTTCTTTTTGTCAAGACCGACCTGTCCGGTGCCCTGAATGAACTGCAGGACGAGGCAGCGCGGCTATCGTGGCTGGCCACGACGGGCGTTCCTTGCGCAGCTGTGCTCGACGTTGTCACTGAAGCGGGAAGGGACTGGCTGCTATTGGGCGAAGTGCCGGGGCAGGATCTCCTGTCATCTCACCTTGCTCCTGCCGAGAAAGTATCCATCATGGCTGATGCAATGCGGCGGCTGCATACGCTTGATCCGGCTACCTGCCCATTCGACCACCAAGCGAAACATCGCATCGAGCGAGCACGTACTCGGATGGAAGCCGGTCTTGTCGATCAGGATGATCTGGACGAAGAGCATCAGGGGCTCGCGCCAGCCGAACTGTTCGCCAGGCTCAAGGCGCGCATGCCCGACGGCGATGATCTCGTCGTGACCCATGGCGATGCCTGCTTGCCGAATATCATGGTGGAAAATGGCCGCTTTTCTGGATTCATCGACTGTGGCCGGCTGGGTGTGGCGGACCGCTATCAGGACATAGCGTTGGCTACCCGTGATATTGCTGAAGAGCTTGGCGGCGAATGGGCTGACCGCTTCCTCGTGCTTTACGGTATCGCCGCTCCCGATTCGCAGCGCATCGCCTTCTATCGCCTTCTTGACGAGTTCTTCTGAGCGGGACTCTGGGGTTCGAAATGACCGACCAAGCGACGCCCAACCTGCCATCACGAGATTTCGATTCCACCGCCGCCTTCTATGAAAGGTTGGGCTTCGGAATCGTTTTCCGGGACGCCGGCTGGATGATCCTCCAGCGCGGGGATCTCATGCTGGAGTTCTTCGCCCACGGGATCTCTGCGGAACAGGCGGTCGAAGGTGCCGATATCATTACGACAGCAACGGCCGACAAGCACAACGCCACGATCCTGAGCGACAATATGATCGGGCCCGGCGTCCACATCAACGGCGTCGGCGGCGACTGCCCAGGCAAGACCGAGATGCACCGCGATATCTTGCTGCGTTCGGATATTTTCGTGGAGTTCCCGCCACAGACCCGGATGATCCCCGATCGTTCAAACATTTGGCAATAAAGTTTCTTAAGATTGAATCCTGTTGCCGGTCTTGCGATGATTATCATATAATTTCTGTTGAATTACGTTAAGCATGTAATAATTAACATGTAATGCATGACGTTATTTATGAGATGGGTTTTTATGATTAGAGTCCCGCAATTATACATTTAATACGCGATAGAAAACAAAATATAGCGCGCAAACTAGGATAAATTATCGCGCGCGGTGTCATCTATGTTACTAGATCGGGCCAATACGCAAACCGCCTCTCCCCGCGCGTTGGCCGATTCATTAATGCAGCTGGCACGACAGGTTTCCCGACTGGAAAGCGGGCAGTGAGCGCAACGCAATTAATGTGAGTTAGCTCACTCATTAGGCACCCCAGGCTTTACACTTTATGCTTCCGGCTCGTATGTTGTGTGGAATTGTGAGCGGATAACAATTTCACACAGGAAACAGCTATGACCATGATTACGCCaagcttGAGCTCCTGCAGGTCACTGGATTTTGGTTTTAGGAATTAGAAATTTTATTGATAGAAGTATTTTACAAATACAAATACATACTAAGGGTTTCTTATATGCTCAACACATGAGCGAAACCCTATAAGAACCCTAATTCCCTTATCTGGGAACTACTCACACATTATTCTGGAGAAAATAGAGAGAGATAGATTTGTAGAGAGAGACTGGTGATTTTTGCGGACTCTATCGACGGATCGGGCTAGAGTTCGTCTTTGGAACCATTCAGATCCTCTTCTGAGATGAGTTTTTGTTCTgcggccgcGTAACAAGTCCCGCCGGTATCGGACATAATGAGGGAAACATTATACAATGTCGGTTTGCTAGCAACAGAGGCGTCAAAATGAACCGGCACATAGGGATTTCCTTCGCAGGCGACAATTATGTGTTTATTAGCTTGAGTAGTTTTATAAGCGCAATTTGGATATTTGCTAGACCCAGTTTCTCGGCAATCGGTGATAGACATGGTGGAGTAGCTTTGGTAGCAATTCGTTTGACCATTCTTACAAGCCACATTCTTTTGTGAACAAACAGCCTGAACATCTGCCAATGACTCGTGGACAAATGTGTTTACAGGCTTACACCTGTCCTTTGTAAGGTTACGTGACTTCATCATCTGGTTACAGTAGTTACTAGAACTggatccTGCTAAACATGTGCTTGTAAAGAGAAGCAAGGAAAGTGAAAGCAAAGATAGAAAAGGTTTGGAAGCCATGGTTCCCGGCCGGGTCAGATCCTCTAGAGTCGATCGAGGTCCTCTCCAAATGAAATGAACTTCCTTATATAGAGGAAGGGTCTTGCGAAGGATAGTGGGATTGTGCGTCATCCCTTACGTCAGTGGAGATATCACATCAATCCACTTGCTTTGAAGACGTGGTTGGAACGTCTTCTTTTTCCACGATGTTCCTCGTGGGTGGGGGTCCATCTTTGGGACCACTGTCGGTAGAGGCATTCTTGAACGATAGCCTTTCCTTTATCGCAATGATGGCATTTGTAGAAGCCATCTTCCTTTTCTACTGTCCTTTCGATGAAGTGACAGATAGCTGGGCAATGGAATCCGAGGAGGTTTCCCGATATTACCCTTTGTTGAAAAGTCTCAATAGCCCTCTGGTCTTCTGAGACTGTATCTTTGATATTCTTGGAGTAGACGAGAGTGTCGTGCTCCACCATGTTGACCTGCAGGTCGACAAGCTTATCGATACCgtcgacCTCGAGGGGGGGCCCGGTACCAAAACCACCCCAGTACATTAAAAACGTCCGCAATGTGTTATTAAGTTGTCTAAGCGTCAATTTGTTTACACCACAATATATCCTGCCACCAGCCAGCCAACAGCTCCCCGACCGGCAGCTCGGCACAAAATCACCACTCGATACAGGCAGCCCATCAGTCCACTAGA
