# Supplementary material for: Plant crude extracts containing oligomeric hemagglutinins protect chickens against highly Pathogenic Avian Influenza Virus after one dose of immunization
Source: Vet Res Commun. 2022 May 28;47(1):191–205. doi: 10.1007/s11259-022-09942-3 (PMC9145123; doi:10.1007/s11259-022-09942-3)
Supplement: Supplementary file 2 — Supplementary file2 (DOCX 14 kb) Table S2. Sequence pair distances [file 11259_2022_9942_MOESM2_ESM.docx]

**Table S2. Sequence pair distances**

| **Divergence** | **Percent Identity** | | | | | |  |
| --- | --- | --- | --- | --- | --- | --- | --- |
|  |  | **1** | **2** | **3** | **4** |  |  |
|  | **1** |  | 93.2 | 94.6 | 92.1 | **1** | **NIBRG-14** |
|  | **2** | 7.2 |  | 98.0 | 98.3 | **2** | **H5-17A384** |
|  | **3** | 5.6 | 2.1 |  | 96.9 | **3** | **H5-A-Hubei** |
|  | **4** | 8.4 | 1.7 | 3.2 |  | **4** | **H5-NAV0292** |
|  |  | **1** | **2** | **3** | **4** |  |  |

Four amino acid H5 sequences of influenza A H5N1 viruses of A/Chicken-DL-NAVET_0292-2013(H5N1) (short name: H5-NAV0292), A/DK/VN/Bacninh/NCVD-17A384/2017 (short name: H5-17A384), A/Vietnam/1194/2004 (H5N1) (short name: NIBRG-14), and A/Hubei/1/2010(H5N1)-PR8-IDCDC-RG30 (short name: H5-A-Hubei) were aligned by using clustal W method and using the Kimura metric. Hemagglutinins from A/Chicken-DL-NAVET_0292-2013(H5N1) had 98.3% deduced hemagglutinin amino acid sequence similarity with A/DK/VN/Bacninh/NCVD-17A384/2017. They were the same clade 2.3.2.1c, while NIBRG-14 was the clade 1, shared 92.1 and 93.2% identity with A/Chicken-DL-NAVET_0292-2013(H5N1) and A/DK/VN/Bacninh/NCVD-17A384/2017, respectively.
