# Supplementary material for: Plant crude extracts containing oligomeric hemagglutinins protect chickens against highly Pathogenic Avian Influenza Virus after one dose of immunization
Source: Vet Res Commun. 2022 May 28;47(1):191–205. doi: 10.1007/s11259-022-09942-3 (PMC9145123; doi:10.1007/s11259-022-09942-3)
Supplement: Supplementary file 3 — Supplementary file3 (DOCX 20 kb) Table S3. Analysis of wild-type H5N1 virus presence three days and ten days after the viral challenge in collected swabs by real-time RT-PCR [file 11259_2022_9942_MOESM3_ESM.docx]

**Table S3. Analysis of wild type H5N1 virus presence three days and ten days after the viral challenge in collected swabs by real-time RT-PCR.**

| **Group** | **Chicken name** | **Realtime RT-PCR** | |
| --- | --- | --- | --- |
|  |  | **3 days after challenge** | **10 days after challenge** |
| **H5 oligomer plant crude extract** | 3 | 29.51 (+) | 31.7 (+) |
|  | 4 | 29.95 (+) | (-)* |
|  | 5 | (-)* | (-)* |
|  | 6 | 37.76 (-) | (-)* |
|  | 7 | 34.81 (+) | (-)* |
|  | 8 | (-)* | (-)* |
|  | 9 | 33.34 (+) | (-)* |
|  | 10 | 33.91 (+) | (-)* |
|  | 11 | (-)* | 29.37 (+) |
|  | 12 | 34.99 (+) | (-)* |
|  | 14 | 32.42 (+) | (-)* |
|  | 15 | (-)* | 30.49 (+) |
|  | **Total** | **7/12 (+)** | **3/12 (+)** |
| **H5 -S*tag trimer plant crude extract** | 31 | 37.04 (-) | (died 6 days after challenge) |
|  | 32 | 34.07 (+) | 36.05 (-) |
|  | 33 | (-)* | 35.98 (-) |
|  | 34 | 31.55 (+) | (-)* |
|  | 36 | 27 (+) | (died 6 days after challenge) |
|  | 37 | 29.71 (+) | 31.64 (+) |
|  | 38 | (-)* | (died 8 days after challenge) |
|  | 39 | 28.96 (+) | (-)* |
|  | 42 | 34.24 (+) | (-)* |
|  | 43 | 34.47 (+) | 32.73 (+) |
|  | 44 | 28.22 (+) | 33.79 (+) |
|  | 45 | 31.26 (+) | 33.61 (+) |
|  | **Total** | **9/12 (+)** | **4/9 (+)** |
| **Wild type plant crude extract** | 46 | 30.1 (+) | (died 5 days after days challenge) |
|  | 47 | 24.22 (+) | died |
|  | 49 | (-)* | died |
|  | 50 | 26.97 (+) | died |
|  | 51 | 23.95 (+) | died |
|  | 52 | 25.16 (+) | (died 4 days after challenge) |
|  | 53 | 30.95 (+) | (died 5 days after challenge) |
|  | 55 | (-)* | (died 4 days after challenge) |
|  | 56 | (-)* | died |
|  | 57 | 25.56 (+) | (died 4 days after challenge |
|  | 59 | 26.07 (+) | died |
|  | 60 | 26.94 (+) | died |
|  | **Total** | **9/12 (+)** | **DIED** |
| **PBS** | 123 | 27.99 (+) | died |
|  | 124 | 30.28 (+) | died |
|  | 125 | 23.85 (+) | died |
|  | 126 | 28.09 (+) | died |
|  | 127 | 32.59 (+) | died |
|  | 128 | 25.95 (+) | died |
|  | 129 | 21.6 (+) | died |
|  | 130 | 21.09 (+) | died |
|  | 132 | 16.86 (+) | died |
|  | 133 | 19.93 (+) | died |
|  | 135 | 23.85 (+) | died |
|  | 136 | 27.63 (+) | died |
|  | **Total** | **12/12 (+)** | **DIED** |
| **Navet-Fluvac2 vaccine (clade 2.3.2.1c)** | 61 | No sample | (-)* |
|  | 62 | 32.31 (+) | (-)* |
|  | 64 | 28.68 (+) | (-)* |
|  | 65 | 37.94 (-) | 34.4 (+) |
|  | 67 | 33.01 (+) | (-)* |
|  | 68 | 38.03 (-) | 33.85 (+) |
|  | 69 | (-)* | (-)* |
|  | 70 | 34.35 (+) | (-)* |
|  | 72 | 34.84 (+) | (-)* |
|  | 73 | 36.6 (-) | 32.63 (+) |
|  | 74 | 32.92 (+) | (-)* |
|  | 75 | 29.97 (+) | (-)* |
|  | **Total** | **7/12 (+)** | **3/12 (+)** |

(+): Ct < 35; (-): Ct>35; (-)*: no Ct value
