# Supplementary material for: Plant crude extracts containing oligomeric hemagglutinins protect chickens against highly Pathogenic Avian Influenza Virus after one dose of immunization
Source: Vet Res Commun. 2022 May 28;47(1):191–205. doi: 10.1007/s11259-022-09942-3 (PMC9145123; doi:10.1007/s11259-022-09942-3)
Supplement: Supplementary file 4 — Supplementary file4 (DOCX 15 kb) Table S4. Analysis of wild-type H5N1 virus presence after housing together experiments after by real-time RT-PCR [file 11259_2022_9942_MOESM4_ESM.docx]

**Table S4. Analysis of wild type H5N1 virus presence after housing together experiment after by real-time RT-PCR.**

| **Group** | **Chicken name** | **Realtime RT-PCR** | |
| --- | --- | --- | --- |
|  |  | **Before housing together** | **10 days after housing together** |
| **H5 oligomer plant crude extract** | 3 | (-)* | (-)* |
|  | 4 | (-)* | (-)* |
|  | 5 | (-)* | (-)* |
|  | 6 | (-)* | (-)* |
|  | 7 | 30.35 (+) | (-)* |
|  | 8 | 31.61 (+) | (-)* |
|  | 9 | (-)* | (-)* |
|  | 10 | (-)* | (-)* |
|  | 11 | 29.37 (+) | 32.9 (+) |
|  | 12 | (-)* | (-)* |
|  | 14 | (-)* | (-)* |
|  | 15 | (-)* | (-)* |
|  | Total | **3/12 (+)** | **1/12 (+)** |
| **Non vaccinated chickens** | 138 | (-)* | (-)* |
|  | 139 | (-)* | (-)* |
|  | 140 | (-)* | (-)* |
|  | 141 | (-)* | (-)* |
|  | 142 | (-)* | (-)* |
|  | 143 | (-)* | (-)* |
|  | 144 | (-)* | (-)* |
|  | 145 | (-)* | 32.65 (+) |
|  | 146 | (-)* | (-)* |
|  | 147 | (-)* | (-)* |
|  | Total | **0/10 (+)** | **1/10 (+)** |

(+): Ct < 35; (-): Ct>35; (-)*: no Ct value
