# Supplementary figures and images for: Plant crude extracts containing oligomeric hemagglutinins protect chickens against highly Pathogenic Avian Influenza Virus after one dose of immunization
Source: Vet Res Commun. 2022 May 28;47(1):191–205. doi: 10.1007/s11259-022-09942-3 (PMC9145123; doi:10.1007/s11259-022-09942-3)

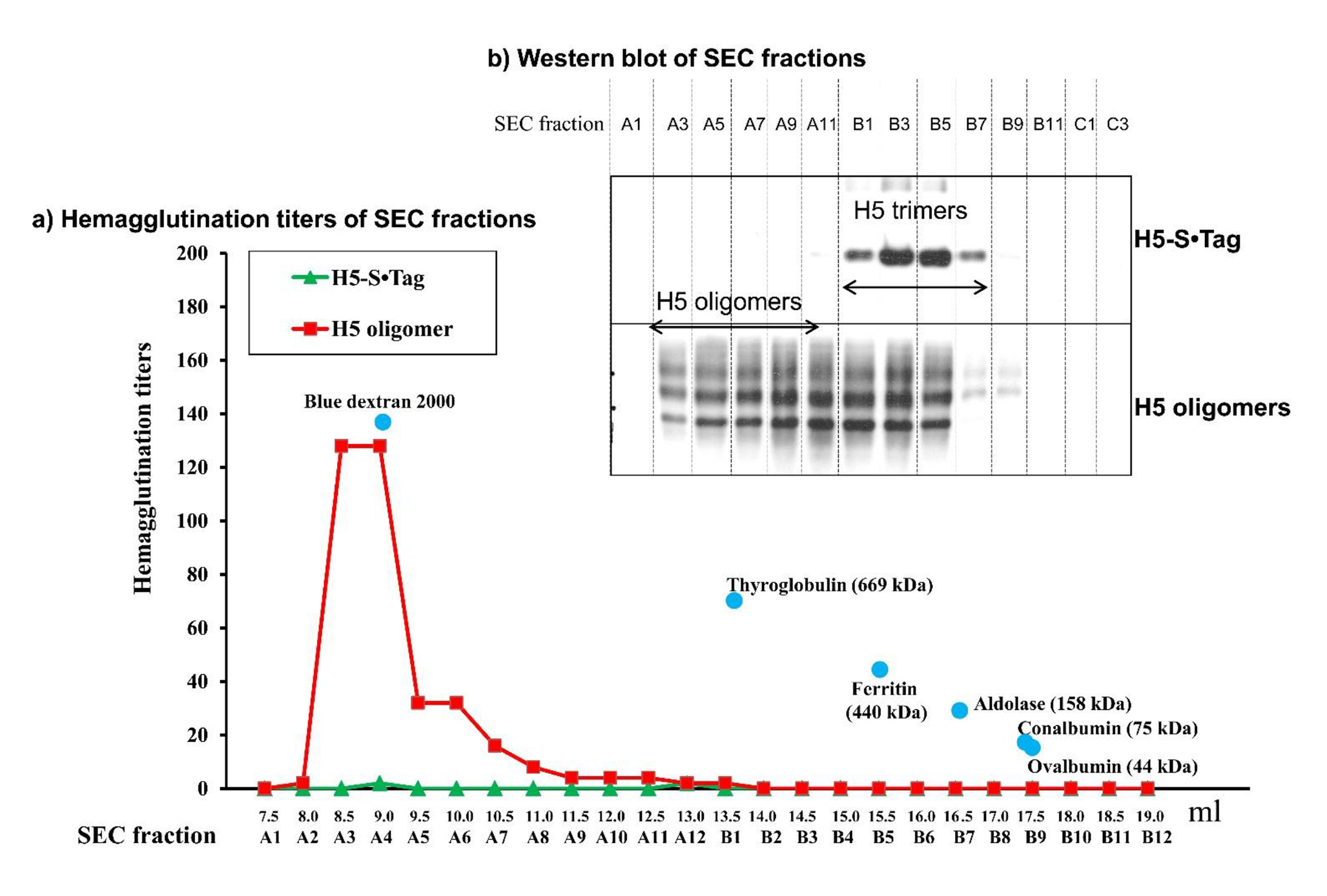

Supplement: Supplementary file 5 — Supplementary file5 (JPG 660 kb) Figure S1. Formation of oligomeric H5 [file 11259_2022_9942_MOESM5_ESM.jpg]
